# Survivin inhibition attenuates EGF-induced epithelial mesenchymal transformation of human RPE cells via the EGFR/MAPK pathway

**DOI:** 10.1371/journal.pone.0309539

**Published:** 2024-08-30

**Authors:** Yusheng Zhu, Teng Li, Sirui Zhou, Guowei Wang, Huihui Zhang, Yong Yin, Tong Wang, Xiaodong Chen

**Affiliations:** 1 Faculty of Life Sciences and medicine, Northwest University, Xi’an, Shaanxi Province, China; 2 First Affiliated Hospital of Northwest University, Northwest University, Xi’an, Shaanxi Province, China; 3 Department of Ophthalmology, Xi’an No.1 Hospital, Xi’an, Shaanxi Province, China; 4 Shaanxi Institute of Ophthalmology, Shaanxi Provincial Key Lab of Ophthalmology, Clinical Research Center for Ophthalmology Diseases of Shaanxi Province, Xi’an, Shaanxi Province, China; 5 Xi’ an Eye Bank, Xi’an No.1 Hospital, Xi’an, Shaanxi Province, China; Manipal Institute of Technology, INDIA

## Abstract

**Purpose:**

The abnormal growth factors–induced epithelial-mesenchymal transition (EMT) in retinal pigment epithelial (RPE) cells was known as a vital pathogenesis of proliferative vitreoretinopathy (PVR). This study aims to explore how survivin inhibition affects EMT induced by epidermal growth factor (EGF) in RPE cells.

**Methods:**

Human primary RPE cells were identified *in vitro*. EMT in RPE cells was induced by EGF. Inhibition of survivin in RPE cells was accomplished through the use of a survivin inhibitor (YM155) and survivin siRNA. The viability, proliferation and migration of RPE cells was detected by methylthiazol tetrazolium assay, bromodeoxyuridine labeling assay, and wound healing assay, respectively. The EGF receptor /mitogen–activated protein kinase (EGFR/MAPK) proteins and EMT-related proteins were measured by western blot and immunofluorescence assay.

**Results:**

EGF induced significant EMT in RPE cells, activated the phosphorylation of EGFR/MAPK signaling proteins, and caused changes to EMT-related proteins. YM155 suppressed RPE cells’ viability, proliferation, and migration; induced the phosphorylation of EGFR, JNK, and P38MAPK; and down regulated EGFR and phosphorylated ERK. YM155 also increased expression of E-cadherin and ZO-1 proteins and reduced expression of N-cadherin, Vimentin, and α-SMA proteins. The EGF-induced increase of RPE cell proliferation and migration was constrained by survivin inhibition. Moreover, survivin inhibition in RPE cells suppressed the EGF-caused phosphorylation of EGFR/MAPK proteins and attenuated the EGF-induced reduction of E-cadherin and ZO-1 proteins and increase of N-cadherin, Vimentin, and α-SMA proteins.

**Conclusions:**

Survivin inhibition attenuates EGF-induced EMT of RPE cells by affecting the EGFR/MAPK signaling pathway. Survivin might be a promising target for preventing PVR.

## Introduction

Proliferative vitreoretinopathy (PVR) is a serious blinding disease, which often occurs after failed surgery of rhegmatogenous retinal detachment and ocular trauma. The retinal pigment epithelium (RPE) cell is a major cell type that is involved in PVR and epithelial-mesenchymal transition (EMT) of RPE cells is identified as an important pathogenic factor of PVR [1]. EMT refers to cells under certain conditions changing to mesenchymal cell morphology from the epithelial cell phenotype. RPE cells are a group of cells containing brown pigment and are located between the choroid and neural retina layer [2]. If the intraocular environment experienced abnormal changes, RPE cells would detach from the retina, diffuse into the vitreous cavity, and then transform into fibroblast cell-like cells, ultimately leading to EMT formation and the development of PVR [3]. Some studies showed that EMT in RPE cells can be caused by abnormal secretion of growth factors including platelet-derived growth factor (PDGF), transforming growth factor-beta (TGFβ),epidermal growth factor (EGF),and et al. [4]. Yet the mechanism of the EMT of RPE cells is not fully clarified and the treatment of PVR remains a huge challenge for researchers.

EGF receptor (EGFR) is a tyrosine kinase receptor widely distributed on the cell membrane, which function is binding to EGF and playing important roles in cell proliferation, migration, and differentiation [5]. There are two main signaling pathways located downstream from the EGFR signaling: Ras/Raf/MEK/MAPK and PI3K/Akt/mTOR [6]. Some studies showed that the EGF/EGFR signaling can contribute to EMT in RPE cells and promote occurrence of PVR [7–9]. In previous study, we demonstrated the EGFR/MAPK signaling was involved in RPE cell survival [10, 11], moreover a recent study showed that EGFR signaling modulates yes-associated protein activation and promotes PVR [12]. However, the specific mechanisms of EGFR-mediated signaling in EMT of RPE cells and PVR remain unclear.

Survivin, a multifunctional antiapoptotic protein belonging to the inhibitor of apoptosis protein family [13], exists in embryonic cells and tumor cells and is rarely expressed in normal cells [14]. Survivin is thought to have a dual biological function in inhibiting apoptosis and regulating the cell cycle [15]. Some studies showed that survivin expressed in RPE cells and contributed to cell survival [16, 17] and that survivin knockdown caused the inhibition of EMT in RPE cells by affecting TGFβ signaling [18]. It has been also found that survivin inhibition attenuates EMT in tumor cells [19] and fibroblasts [20]. Sepantronium bromide YM155, an effective survivin inhibitor, is thought to significantly suppress activity of survivin promoter. YM155 plays valuable roles in cell apoptosis and cancer therapy [21, 22]. Some studies showed that YM155 can inhibit cell proliferation, migration, and EMT through the TGF-β pathway in RPE cell lines [18]. Our previous studies showed that YM155 suppressed the survival of RPE cell lines through EGFR/MAPK proteins [23]. Nevertheless, the mechanism of survivin inhibition affecting EMT has not been adequately elucidated.

In present study, the effects of survivin inhibition on EGF-induced EMT of RPE cells were investigated. Our findings demonstrated that survivin inhibition by using YM155 and survivin-siRNA transfection significantly attenuated EGF-induced EMT in RPE cells. In conclusion, our study suggest survivin might be a potential target for treating PVR.

## Materials and methods

### Reagents

EGF was purchased from Peprotech Inc. (Rocky Hill, NJ, USA) and YM155 and 5-Bromodeoxyuridinc (BrdU) from Selleck Chemicals LLC (Houston, TX, USA). Thiazolyl Blue Tetrazolium Bromide (MTT), fetal bovine serum (FBS), and FluoroShield™ with 4,6-diamino-2-phenyl indole (DAPI) were from Sigma-Aldrich (St. Louis, MO, USA). Dulbecco’s Modified Eagle Medium: Nutrient Mixture F-12 (DMEM/F12) was from Thermo Fisher Scientific (Waltham, Massachusetts, USA). Small interfering RNA (SiRNA) targeting the survivin, control siRNA, and bovine serum albumin were Santa Cruz Biotechnology Inc. (Dallas, Texas, USA).

### Primary human RPE and ARPE-19 cell line cell culture

Primary human RPE cells were isolated from ocular tissues, which were generously donated by volunteer donors to a Xi’an eye bank in China after a keratoplasty between January 1, 2021 and December 31, 2023. An informed written agreement was obtained from volunteer donors themselves or their family members. All the procedures were approved by the Ethics Committee of Xi’an No.1 Hospital according to the tenets of the Declaration of Helsinki. The extra tissues of sclera were removed and trypsin containing 0.25% ethylene diamine tetraacetic acid was used to digested and separated the RPE cells in at 37°C for 1–1.5h. In the next step, the RPE cells were centrifuged at 1200 rpm for 5 minutes and cultured in DMEM/F12 medium containing 20% fetal bovine serum and 1% penicillin-streptomycin solution. 4–6 passages of RPE cells were then selected for the further experiments. ARPE-19 cell line came from American Type Culture Collection (Manassas, VA, USA) and were cultured with DMEM/F12 medium contained 10% FBS at 37°C in a incubator with 5% carbon dioxide. ARPE-19 cells were used as a positive control.

### SiRNA transfection

The survivin siRNA and control siRNA were transfected into RPE cells through Lipofectamine 2000 and Opti-MEM^TM^ I Reduced Serum Medium (Thermo Fisher Scientific, Massachusetts, USA) according to operation guidelines. Transfection efficacy was analyzed by western blot through assessing expression of survivin protein.

### Cell viability assay

After treatment with different reagents, the RPE cells in a 96-wells plate were incubated in the DMEM/F12 medium containing the MTT solution(50μg/mL) for 4h. Then, the DMEM/F12 medium was replaced with 150 μl of dimethyl sulfoxide solution(DMSO). When the RPE cells are dissolved by DMSO, the absorbance value representing cell viability was detected at 570nm by using a micro plate absorbance reader (ELx808IU, Gene company limited, HK, China).

### BrdU labeling assay

Cell proliferation was detected by the BrdU labeling assay. After treatment with different reagents, The RPE cells in 12-well plates were incubated in a medium with 30 μmol/L of BrdU for 4 h and rinsed twice with phosphate buffer saline solution(PBS). The 4% paraformaldehyde (PFA) was used to fix cells for 30 minutes. After rinsing twice by PBS, the RPE cells were treated by 500μl of hydrochloric acid (2mol/L) for acidification and blocked by 5% BSA for 1 hour. Then, cells were rinsed thrice in PBS and incubated with a BrdU antibody (Table 1) for 2 hours. Next, the RPE cells were rinsed twice and incubated in fluorescence secondary antibody diluents (1:1000) in the dark for 1 hour. At last, the RPE cells were sealed by FluoroShield™ with DAPI and detected through fluorescence microscope (IX73, Olympus, Japan).

**Table 1:** Primary antibodies used for immunodetection.

Name	Species	Manufacturer	Product number	Application	Dilution
pEGFR	Rabbit	Cell Signaling	3777	WB, IF	1:1000(WB), 1:100(IF)
EGFR	Rabbit	Cell Signaling	4267	WB, IF	1:1000(WB), 1:100(IF)
pP38MAPK	Rabbit	Cell Signaling	4511	WB	1:1000
P38MAPK	Rabbit	Cell Signaling	9212	WB	1:1000
pJNK	Rabbit	Cell Signaling	4668	WB	1:800
JNK	Rabbit	Cell Signaling	9252	WB	1:1000
pERK	Rabbit	Cell Signaling	4370	WB	1:1000
ERK	Rabbit	Cell Signaling	9102	WB	1:1000
E-cadherin	Rabbit	Cell Signaling	3195	WB,IF	1:800(WB), 1:200(IF)
ZO-1	Rabbit	Cell Signaling	13663	WB,IF	1:1000(WB), 1:200(IF)
N-cadherin	Rabbit	Cell Signaling	13116	WB,IF	1:1000(WB), 1:200(IF)
Vimentin	Rabbit	Cell Signaling	5741	WB	1:1000
α-SMA	Rabbit	Cell Signaling	19245	WB, IF	1:1000(WB), 1:200(IF)
BrdU	Mouse	Cell Signaling	5292	IF	1:1000
β-actin	Mouse	Thermo	MA5-11869	WB	1:500

### Cell migration assay

When the RPE cells grew to 100% confluence, scratches of “wound injury” were performed by a 200 μl tip. Then the RPE cells were treated by different reagents, and wound-healing images were observed by a inverted phase contrast microscope. Finally, the relative distance of cell migration was quantified by Image J software.

### Western blot

After treating with different reagents, the RPE cells were softly washed twice in PBS and cracked by a 1.5×SDS (sodium dodecyl sulfonate) buffer. The sample proteins were then denatured in a metallic bath at 100°C for 10min, separated by 10% SDS-polyacrylamide-tricine gels. Next, sample proteins were transferred to the polyvinylidene fluoride (PVDF) membrane, blocked in 5% defatted milk solution for 60 minutes, and rinsed thrice in PBST. Then PVDF was incubated in primary antibody solution (Table 1) at 4°C overnight. The PVDF membrane was rinsed thrice in PBST, and was combined with secondary antibodies for 2 hours at room temperature. After being washed thrice in PBST, the PVDF membrane were soaked in a chemiluminescence solution, and the proteins were detected by a gel documentation system (G:Box Chemi-XR, Gene company limited, HK, China) and analyzed with Image J software. β-actin was the internal reference control.

Primary antibodies with different dilution ratio were used in western blot and immunofluorescence.

### Immunofluorescence assay

The RPE cells were cultured on sterile glass slides, treated with different reagents, and fixed by 4% PFA. Next, cells were rinsed thrice, permeabilized in a 0.1% Triton X-100 solution for 2 minutes, and rinsed thrice again. Then, cells were blocked with 5% BSA for 1 hour, incubated in a primary antibody diluent for 2 hours, and rinsed thrice again. The RPE cells were incubated in a fluorescent secondary antibody diluent (1:1000) for 1 hour, rinsed thrice, sealed with FluoroShield™ containing DAPI. Finally, cells were observed by the fluorescence microscope(IX73, Olympus, Japan).

### Statistical analysis

All data from at least three times independent experiments were presented as mean±SEM and analyzed by GraphPad Prism 8.0 software (GraphPad Software, La Jolla, CA, USA). The unpaired T-test was used to analyzed the difference between two groups. The one-way ANOVA with the post hoc Dunnett test was used to analyzed the difference among more groups. *P* < 0.05 was considered statistically significance.

## Results

### The expression of EGFR and survivin in RPE cells

**Fig 1:**
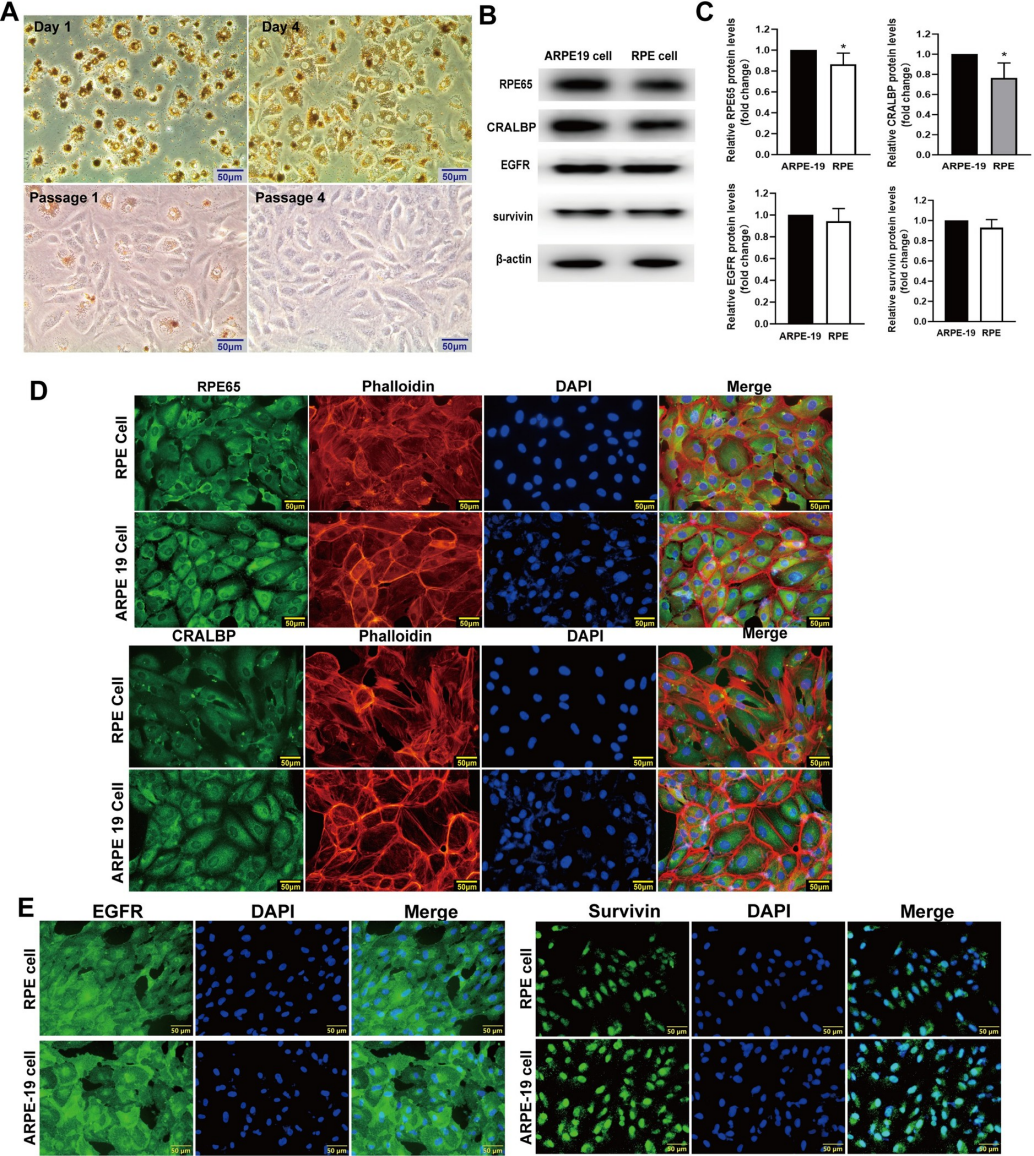
The identification of human primary RPE cells. (**A**) Images of morphology of primary RPE cells at 1 day and 4 days of being isolated from ocular tissue, and images of morphology of first passage and fourth passage RPE cells. (**B**) During the fourth passage, the RPE65 and CRALBP of ARPE-19 cells and the fourth passage human RPE cells were determined by western blot. (**C**) The quantitative data of RPE65 and CRALBP proteins in B,n = 3, *P < 0.05. (**D**) During the fourth passage, the RPE65 and CRALBP proteins in ARPE-19 cells and the fourth passage human RPE cells were determined by immunofluorescence. Scale bar = 50 μm. (**E**) EGFR and survivin were detected by immunofluorescence in primary RPE cells and ARPE-19 cells. Scale bar = 50 μm.

In order to verify the expression of EGFR and survivin proteins in human RPE cells, we cultured primary human RPE cells and detected expression of EGFR and survivin proteins in human primary RPE cells and ARPE-19 cell lines by immunoblotting and immunofluorescence. The images of phase contrast microscope showed that the morphology of the primary RPE cells was round and that cytoplasm contains a large amount of brown-black pigment after one day of isolation from human ocular tissues. Four days later, the morphology of the RPE cells was shaped like an irregular hexagon, and the brown-black pigment in the cytoplasm began to decrease. After the first passage, the cytoplastic brown-black pigment in the RPE cells significantly reduced, and the RPE cells were distributed like flat and irregular polygons. In fourth passage, the cytoplastic brown-black pigment had completely disappeared (Fig 1A). The marker proteins of the RPE cells, including RPE65 and CRALBP, were determined by western blot. The results showed that the expression of the RPE65 and CRALBP proteins in the fourth passage RPE cells was fewer compared to the ARPE-19 cells At the same time, primary RPE cells and ARPE-19 cells both expressed EGFR and survivin proteins (Fig 1B and 1C). The immunofluorescence assay showed that during the fourth passage, the RPE cells significantly expressed RPE65 and CRALBP protein, and the cells’ nuclei were uniform in size (Fig 1D). Additionally, immunofluorescence results indicated that primary RPE cell and ARPE-19 cell expressed EGFR and survivin proteins (Fig 1E). These results showed that both EGFR and survivin proteins were expressed human RPE cells.

### EGF induced EMT of RPE cells

**Fig 2:**
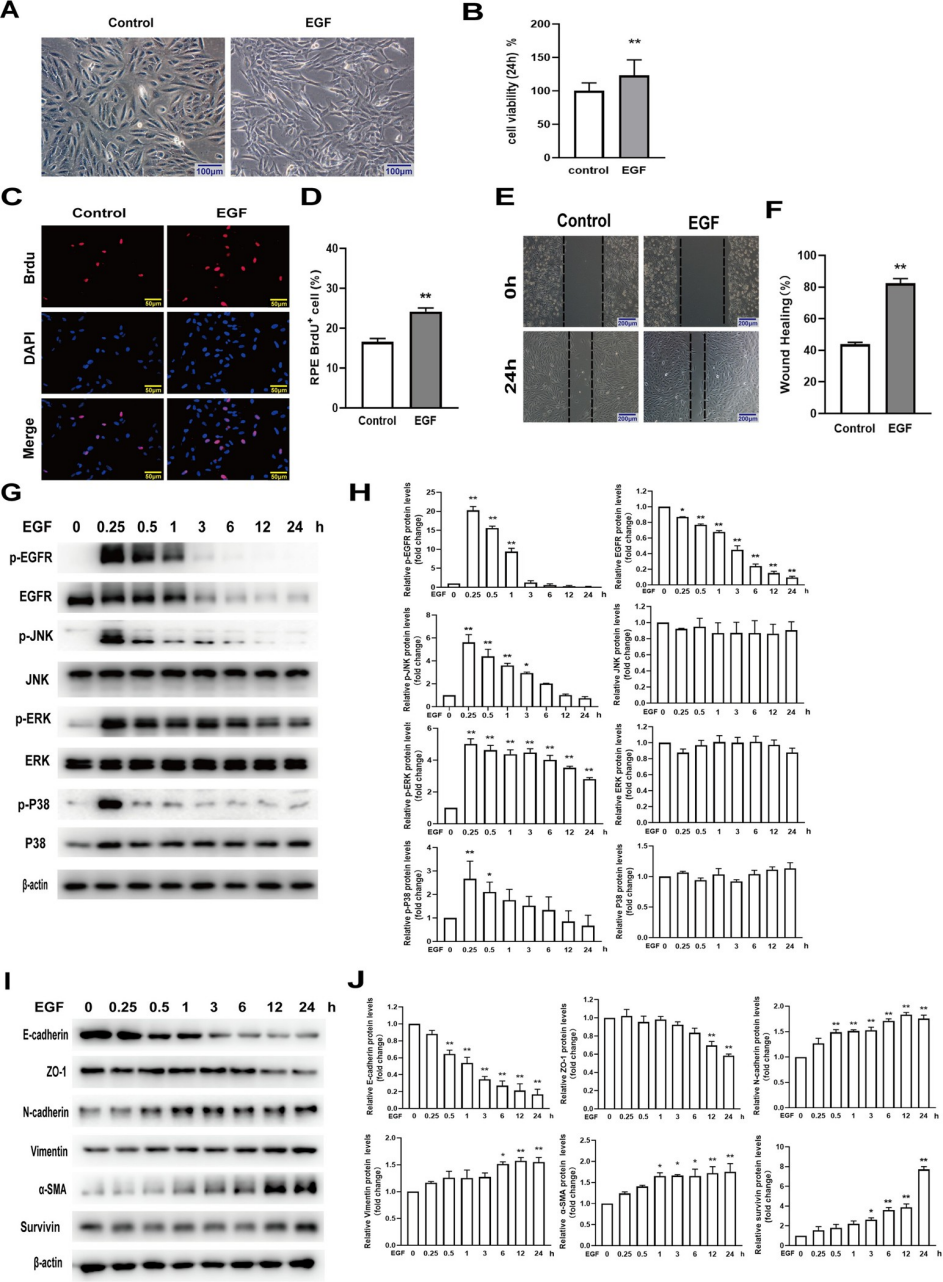
EGF induced EMT in RPE cells. RPE cells were treated by 100 ng/ml of EGF for 24h. (A) The morphology of cells was observed by a inverted phase contrast microscope. (B) Cellular viability was tested by the MTT assay, n = 8. (C, D) Cellular proliferation was determined and analyzed by the BrdU labeling assay with a fluorescence microscope, n = 3. (E,F)Cellular migration was detected and analyzed by the wound-healing assay, n = 3. RPE cells were treated by EGF (100 ng/ml) for 0.25, 0.5, 1, 3, 6, 12, and 24h. Nonphosphorylated and phosphorylated EGFR,JNK, ERK, and P38MAPK (G,H), and E-cadherin, ZO-1, N-cadherin, α-SMA and Vimentin proteins (I,J) were determined by western blot, n = 3. *P < 0.05, **P < 0.01.

To determine whether EGF could induce EMT of RPE cells via the EGFR /MAPK pathway, we observed the effect of 100 ng/mL of EGF on human RPE cells. Our results showed that treatment with EGF(100 ng/mL) induced RPE cells to transform into a spindle from flat and irregular polygons (Fig 2A). The MTT results suggested that treatment with 100 ng/ml EGF increased the RPE cell viability by 24% compared to the control (Fig 2B). RPE cell proliferation capacity was tested by the BrdU labeling assay. Our results showed the BrdU staining positive RPE cells was 16.6% in the control group, while was 24.1% in the EGF group. The BrdU staining positive RPE cells increased by 7.5% compared to the control group (Fig 2C and 2D). The results of wound-healing assay showed treatment with 100 ng/mL EGF increased RPE cell migration by 38.5% compared to the control group (Fig 2E and 2F). The western blot assay showed, with the extension of treatment time, that EGF activated the phosphorylation of EGFR, JNK, ERK and P38MAPK (Fig 2G and 2H), decreased the expression level of E-cadherin and ZO-1 proteins, meanwhile increased the expression level of N-cadherin, Vimentin, and α-SMA proteins (Fig 2I and 2J). These results proved that EGF significantly induced the EMT of human primary RPE cells.

### Effects of YM155 on RPE cells

**Fig 3:**
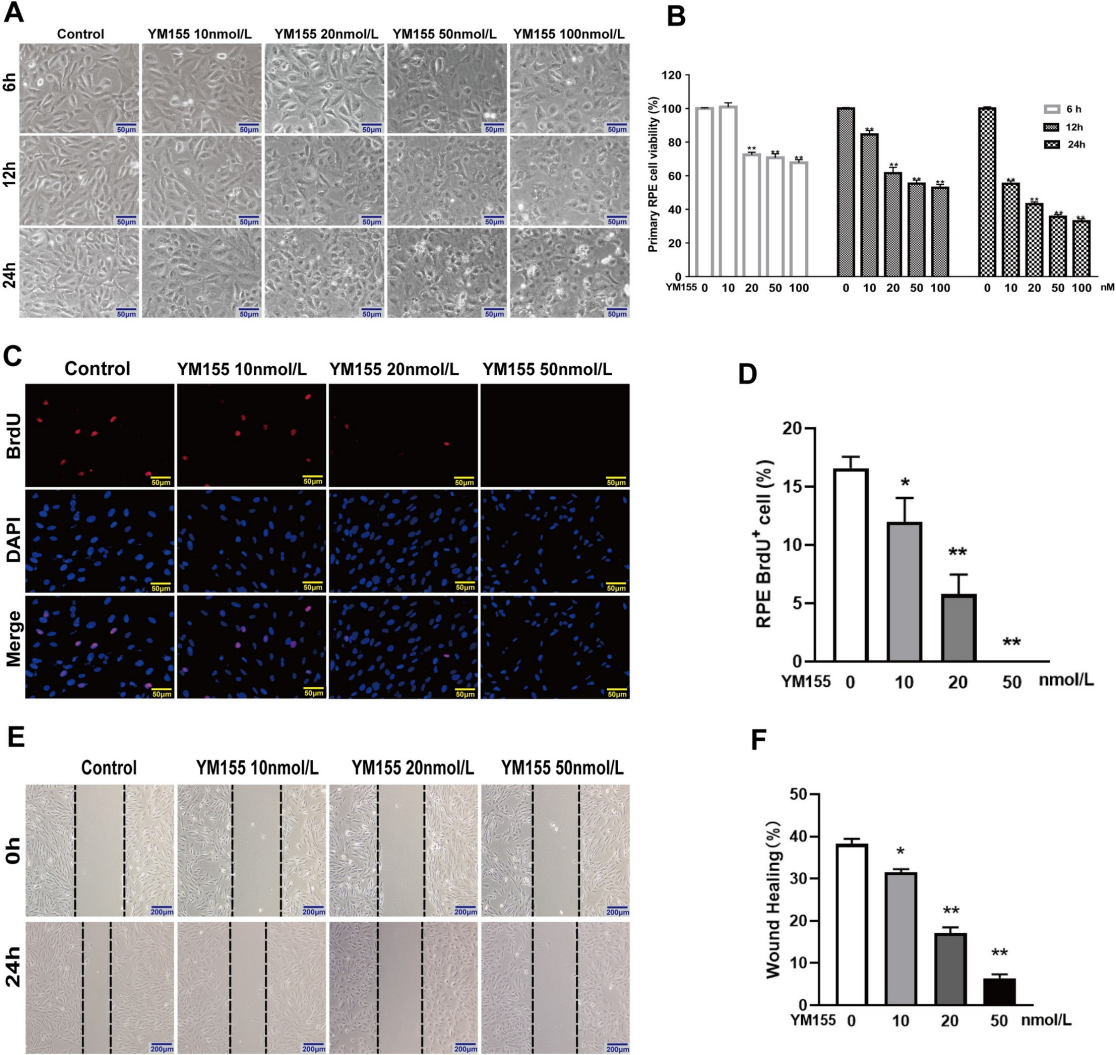
Effects of YM155 on the morphology, viability, proliferation, and migration of RPE cell. Treatment with different doses of YM155 for 6, 12 and 24h. (A)The morphology of RPE cells was observed by a phase-contrast microscope, scale bar = 50 μm. (B) RPE cells’ viability was tested by the MTT assay. Treatment with different concentrations of YM155 for 24h. (C, D)RPE cells’ proliferation was detected and analyzed by the BrdU labeling assay with fluorescence microscope, scale bar:50 μm, n = 3. (E,F) RPE cells’ migration was analyzed by the wound-healing assay, scale bar:200 μm, n = 3. (*P < 0.05, **P < 0.01).

**Fig 4:**
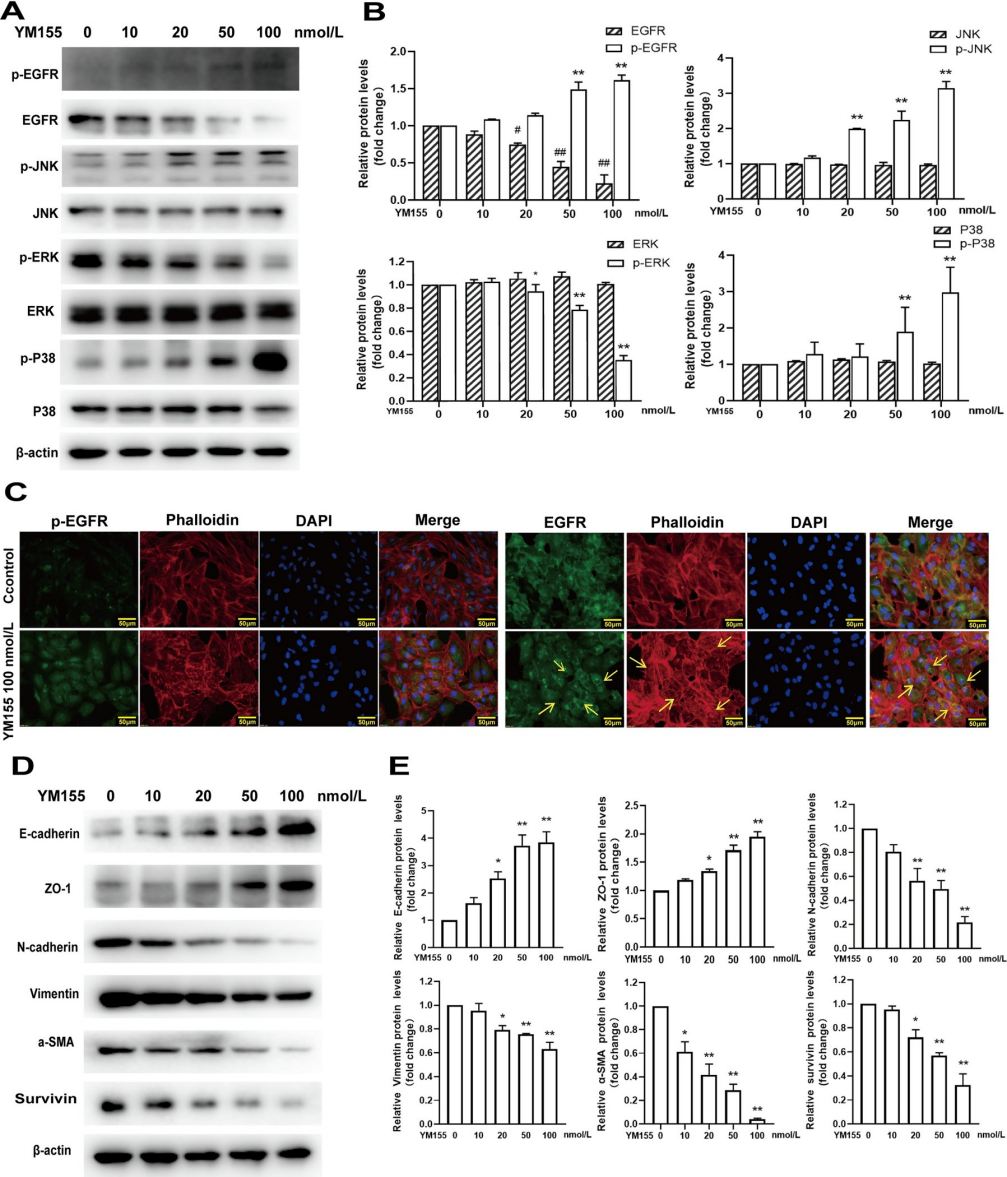
Effects of YM155 on EGFR/MAPK proteins and EMT-related proteins in RPE cell. (A,B)RPE cells were treated with YM155 (0, 10, 20, 50 and 100 nmol/L) for 12 hours. Total and phosphorylated EGFR, JNK, ERK and P38MAPK proteins were determined and analyzed by western blot, n = 3. (C)EGFR, F-actin, and the cellular nucleus in RPE cells were determined by immunofluorescence staining after treatment with YM155 (50 nmol/L) for 12 hours. (D, E)When the RPE cells were treated with YM155 (0, 10, 20, 50, and 100 nmol/L) for 24 hours, E-cadherin, ZO-1, N-cadherin, a-SMA, Vimentin, and the survivin proteins were determined and analyzed by western blot, n = 3. *P < 0.05, **P < 0.01.

To explore the mechanism of survivin inhibition in EMT of RPE cells, we measured the effect of survivin inhibitor YM155 on cell viabiltity, proliferation, and migration of RPE cells, EGFR/MAPK signaling pathway proteins, and EMT-related proteins. The observations from the phase-contrast microscope showed that YM155 caused the RPE cells to become more rounded, and their intercellular spaces also became larger (Fig 3A). YM155 significantly inhibited RPE cells’ viability in a concentration-dependent and time-dependent manners (Fig 3B). The BrdU labeling assay showed that YM155 significantly decreased BrdU staining positive RPE cells(Fig 3C and 3D). The wound-healing assay showed that treatment with YM155 for 24h significantly inhibited the cellular migration distance (Fig 3E and 3F). When the RPE cells were treated by YM155, with an increasing concentration of YM155, the non-phosphorylated EGFR and phosphorylated ERK proteins gradually decreased; meanwhile, the phosphorylated EGFR, JNK, and P38MAPK proteins gradually increased, but the non-phosphorylated ERK, JNK, and P38 proteins did not change significantly (Fig 4A and 4B). Immunofluorescence staining assay indicated YM155 induced the translocation of the EGFR proteins from membrane to cytoplasm and the destruction of F-actin (Fig 4C). YM155 also can result in remarkable change of EMT-related proteins including up-regulation of E-cadherin and ZO-1 proteins, and down-regulation of N-cadherin, a-SMA, and vimentin proteins. In addition, YM155 significantly decreased the expression levels of survivin protein (Fig 4D and 4E). These data indicated that survivin inhibitor YM155 inhibited RPE cells’ viability, proliferation, and migration, and affected EGFR/MAPK signaling pathway proteins and EMT-related proteins.

### YM155 attenuates the EGF-induced EMT in RPE cells

**Fig 5:**
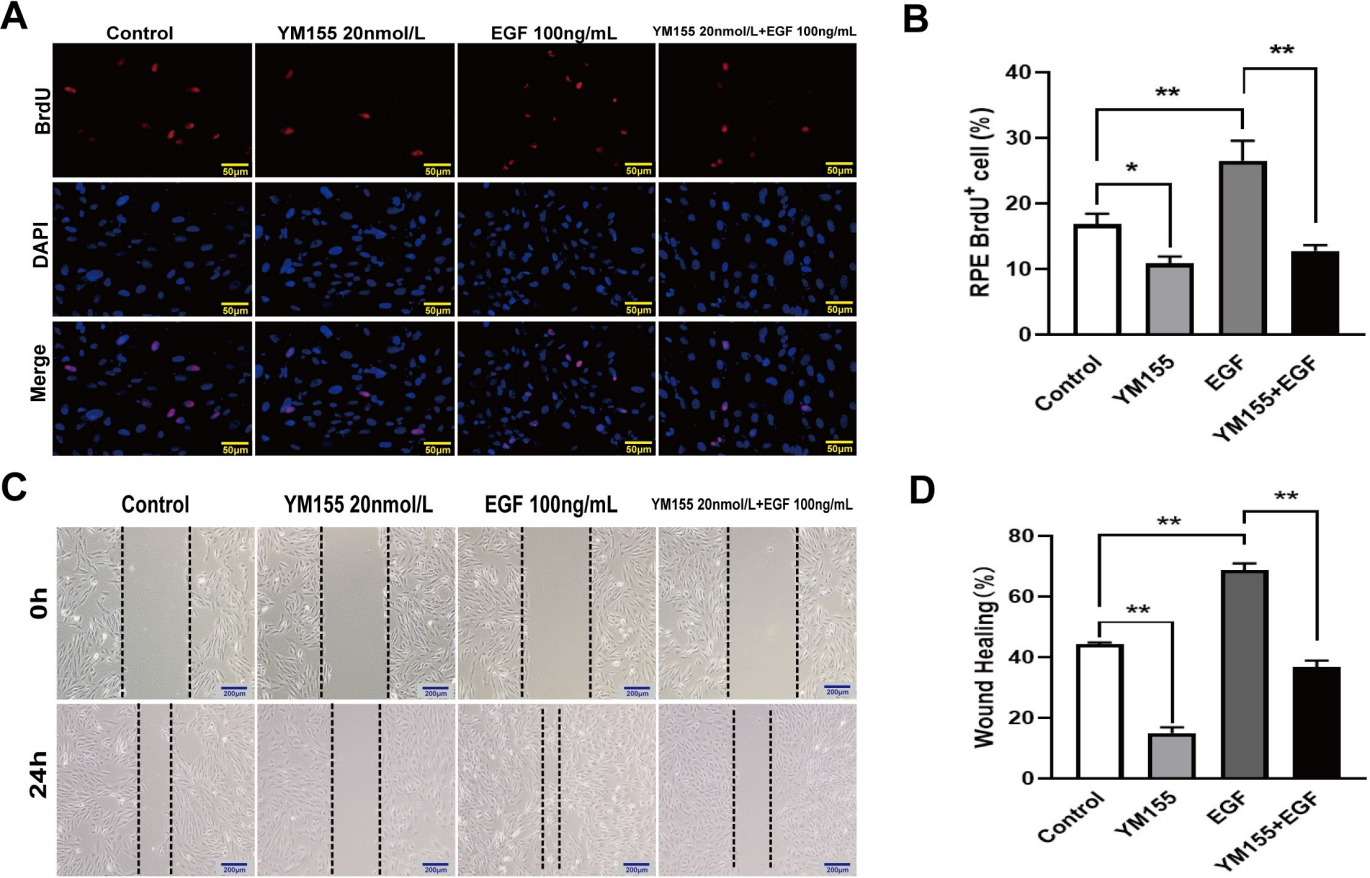
YM155 inhibited EGF-induced RPE cells’ proliferation and migration. The RPE cells were pretreated with 20 nmol/L of YM155 for 12 hours and were treated with EGF (100 ng/mL) for 24 hours. (A, B) Cellular proliferation was labeled with BrdU and analyzed by immunofluorescence assay, scale bar :50 μm, n = 3. (C, D) Cellular migration was tested and analyzed by the wound-healing assay, scale bar: 200 μm, n = 3. *P < 0.05, **P < 0.01.

To clarify whether survivin inhibitor YM155 can inhibit EGF-induced EMT in RPE cells, we observed the effect of pretreatment with 20 nmol/L of YM155 on treatment with EGF (100 ng/mL) in RPE cells. The BrdU labeling assay showed that the surviving inhibitor YM155 significantly inhibited EGF-induced increased of BrdU labeling positive RPE cells (Fig 5A). The BrdU labeling positive RPE cells was reduced by 6% in the YM155 group, and increased by 9.7% in the EGF group compared to the control group. Compared to the EGF treatment group, the BrdU labeling positive RPE cells were reduced by 13.7% in the YM155 combined with EGF group (Fig 5B). The wound-healing assay showed that YM155 significantly inhibited EGF-induced increased of migration of RPE cells (Fig 5C). The RPE cells’ migration distance decreased by 29% in the treatment with YM155 group, whereas increased by 24% in EGF group compared to the control group. The RPE cells’ migration distance decreased by 31% in the YM155 combined with EGF group compared to the EGF treatment group (Fig 5D).

**Fig 6:**
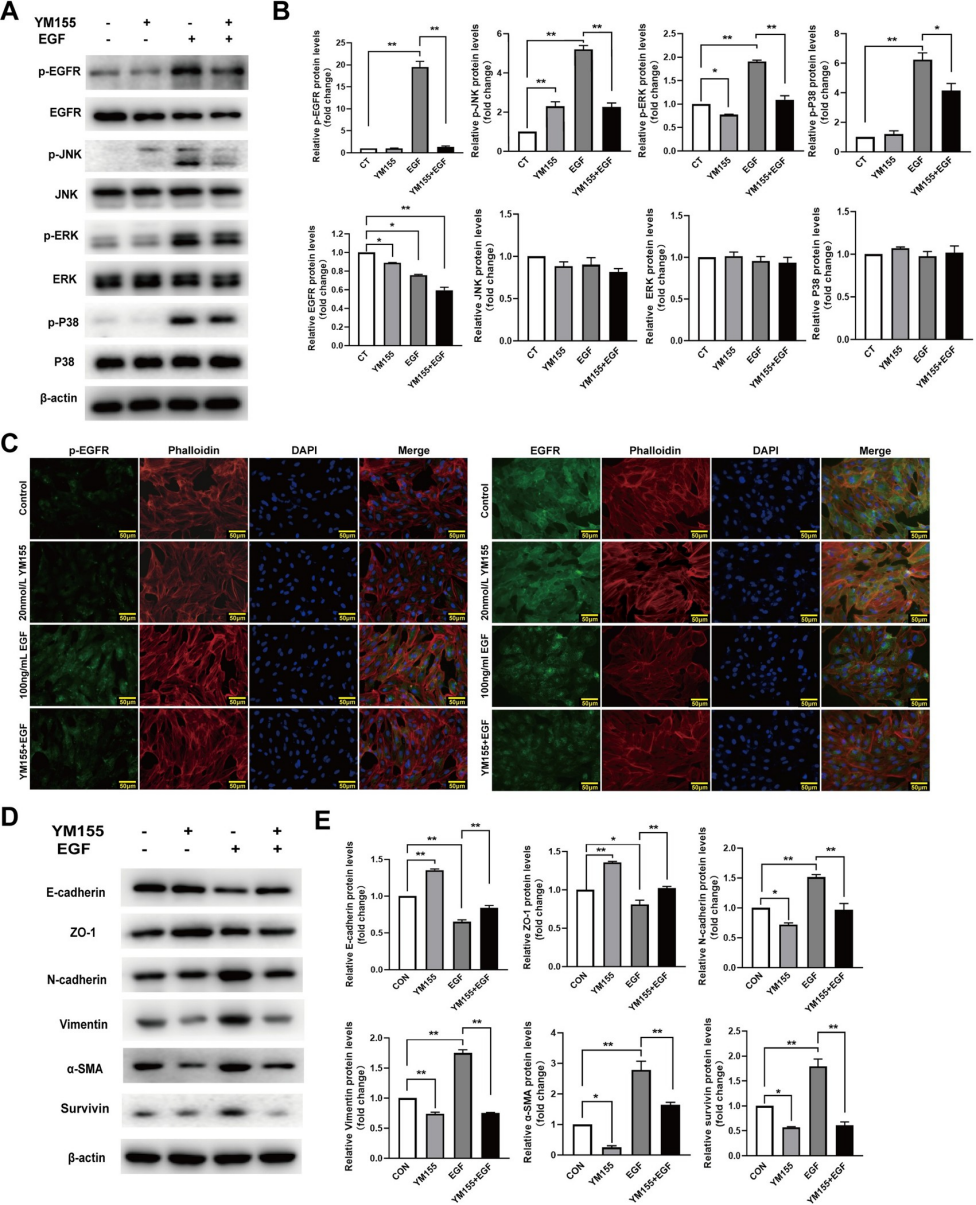
Effects of YM155 on EGF-induced EGFR/MAPK proteins and EMT-related proteins in RPE cells. The RPE cells were pretreated with 20 nmol/L of YM155 for 12 hours and treated with 100 ng/ml of EGF for 15 minutes. (A,B)The phosphorylated and nonphosphorylated EGFR, JNK, ERK, and P38MAPK were detected and analyzed by western blot, n = 3. (C) EGFR and phosphorylated EGFR were determined by immunofluorescence assay, scale bar: 50 μm. (D,E) RPE cells were pretreated with 20 nmol/L of YM155 for 12 hours and treated with 100 ng/mL of EGF for 24 hours. E-cadherin, ZO-1, N-cadherin, a-SMA, Vimentin,and survivin were detected and analyzed by western blot, n = 3. *P < 0.05, **P < 0.01.

The YM155 obviously suppressed EGF-induced phosphorylation of EGFR, JNK, ERK, and P38 MAPK (Fig 6A and 6B). The immunofluorescence assay showed YM155 attenuated EGF-induced EGFR transposition from membrane to cytoplasm. The EGF-induced increase of phosphorylated EGFR expression was inhibited by pretreatment with YM155 (Fig 6C). Pretreatment with YM155 attenuated EGF-induced down-regulation of E-cadherin and ZO-1 proteins and up-regulation of N-cadherin, α-SMA, and vimentin proteins. Furthermore, EGF-induced up-regulation of survivin protein was inhibited by preconditioning with YM155 (Fig 6D and 6E). These results showed that YM155 attenuated the EGF-induced EMT in RPE cells.

### Survivin knockdown affects EMT induced by EGF in RPE cells

**Fig 7:**
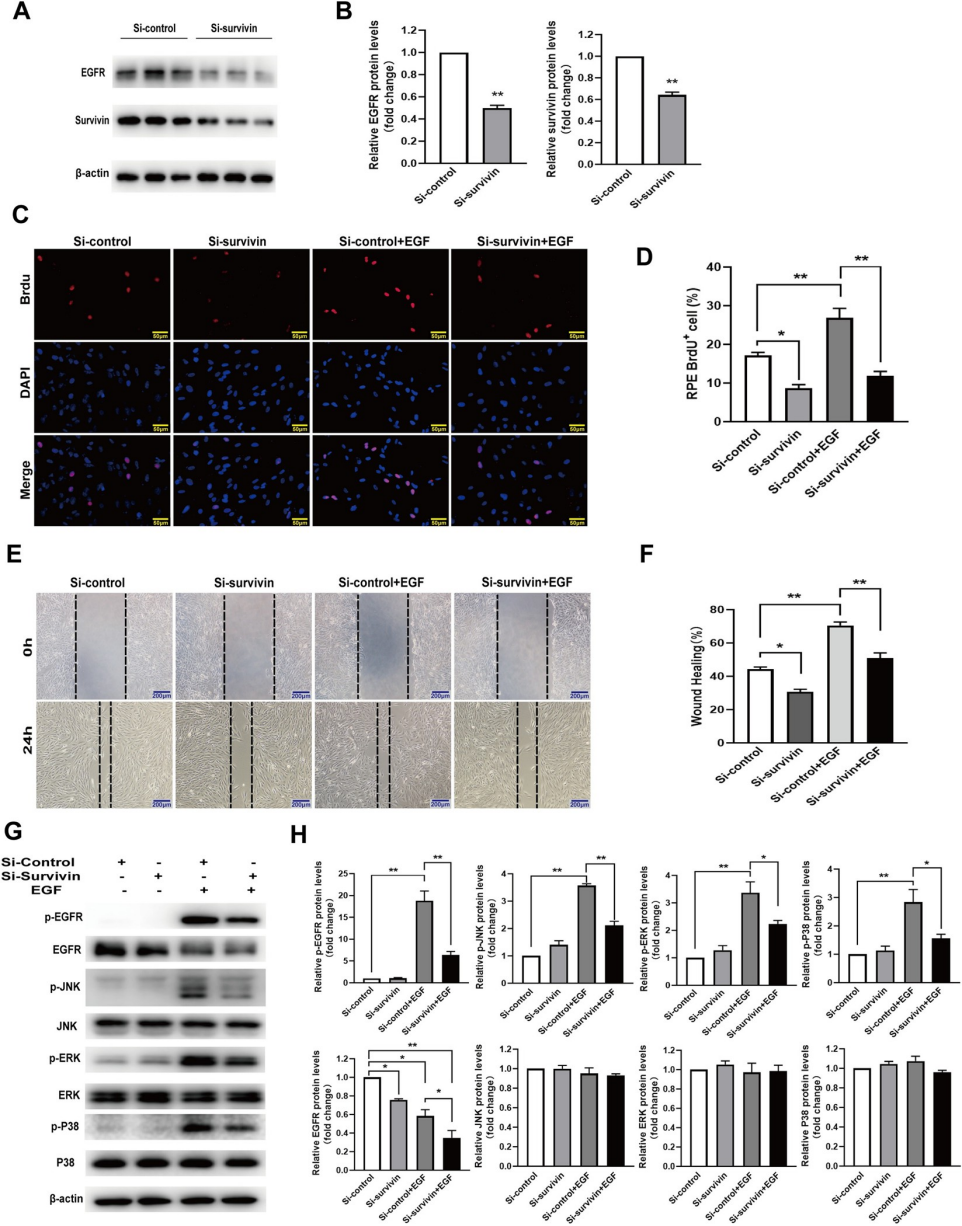
Survivin knockdown suppressed EGF-induced RPE cells’ proliferation and migration by EGFR/MAPK proteins. (A, B)The RPE cells were transfected by the survivin-siRNA, and the survivin and EGFR proteins were detected and analyzed by western blot, n = 3,. The RPE cells were treated with or without EGF (100ng/ml) for 24h after transfection with survivin-siRNA; (C, D)the proliferation of RPE cells was analyzed by the BrdU labeling assay through fluorescence microscope, scale bar:50 μm, n = 3; (E, F)the migration of the RPE cells was analyzed by the wound-healing assay, scale bar: 200 μm, n = 3. The RPE cells were treated by EGF (100 ng/ml) for 15min after transfection with survivin-siRNA, (G, H) non-phosphorylated and phosphorylated EGFR, JNK, ERK, and P38 MAPK proteins were determined and analyzed by western blot, n = 3. *P < 0.05, **P < 0.01.

**Fig 8:**
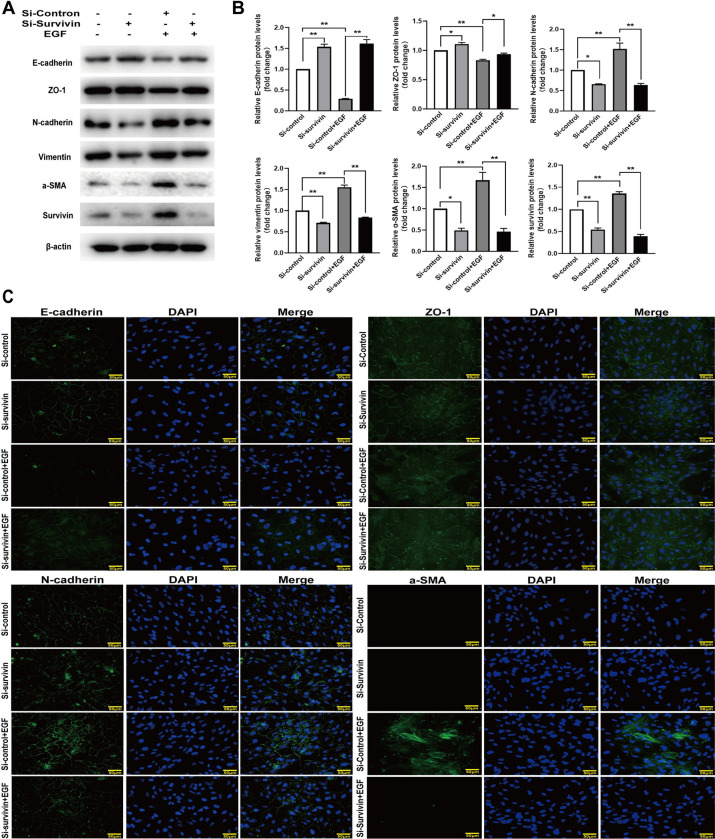
Survivin knockdown attenuated EGF-induced changes of EMT-related proteins in RPE cells. RPE cells were treated with or without EGF (100 ng/ml) for 24h after transfected with survivin-siRNA. (A, B)E-cadherin, ZO-1, N-cadherin, a-SMA, Vimentin, and survivin proteins were detected and analyzed by western blot, n = 3; (C)E-cadherin, ZO-1, N-cadherin, and a-SMA proteins were determined by the immunofluorescence staining assay, scale bar:50 μm. *P < 0.05, **P < 0.01.

In order to further verify the effect of survivin inhibition on EGF-induced EMT in RPE cells, survivin knockdown in RPE cells was achieved by transfecting survivin siRNA, RPE cells’ proliferation and migration, EGFR/MAPK signaling pathway proteins and EMT-related proteins were detected. Western blot results demonstrated that the transfection of survivin siRNA resulted in a significant reduction of survivin protein levels and also down regulated the expression levels of EGFR protein in RPE cells (Fig 7A and 7B). The BrdU labeling assay showed the BrdU^+^ cells were 17.1% in the control group, 8.7% in the survivin knockdown group, 26.9% in the treatment with EGF group, and 11.7% in the survivin knockdown combined with EGF group,respectively.The BrdU^+^ RPE cells were decreased by 8.5% in the survivin knockdown group and increased by 9.7% in the treatment with EGF group compared to the control group. Moreover, compared to the EGF treatment group, BrdU^+^ RPE cells were reduced by 15% in the survivin knockdown combined with EGF group (Fig 7C and 7D). The wound-healing assay indicated that the RPE cells’ migration distance was 44.3%, 30.8%, 70.6%, and 50.9% of the scratch in the control group, in the survivin knockdown group, in the treatment with EGF group, and in the survivin knockdown combined with EGF group, respectively. Compared to the control group, survivin knockdown reduced migration of RPE cells by 13.5%,while EGF increased migration of RPE cells by 26.3%. Compared to the EGF treatment group, survivin knockdown combined with EGF decreased the migration distance of the RPE cells by 19.7% (Fig 7E and 7F). Our western blot assay indicated that EGF obviously induced an increase of phosphorylated EGFR, JNK, ERK, and P38MAPK proteins, whereas the EGF-induced increase of phosphorylated EGFR, JNK, ERK, and P38MAPK proteins was inhibited by survivin knockdown (Fig 7G and 7H). In addition, the survivin knockdown increased the expression of E-cadherin and ZO-1 proteins but decreased the expression of N-cadherin, a-SMA, and vimentin proteins. EGF-induced a reduction of E-cadherin and ZO-1 proteins and an increase of N-cadherin, α-SMA, and vimentin proteins were attenuated survivin knockdown. Furthermore, survivin knockdown suppressed the EGF inducing expression of survivin protein (Fig 8A and 8B). The immunofluorescence staining assay also indicated similar results in that survivin knockdown affected EGF-induced changes of RPE cell morphology, attenuated an EGF-induced reduction of E-cadherin and ZO-1 proteins and an increase of N-cadherin and α-SMA proteins (Fig 8C). These data showed that survivin knockdown obviously inhibit EGF-induced EMT in RPE cells.

## Discussion

PVR is a serious blinding vitreoretinal fibrosis disease, and EMT of RPE cells is considered as a vital pathological mechanism of it [24]. When the EMT occurred, RPE cells lose the connective properties of epithelial cells and develop mesenchymal cell phenotypes [25]. Some cytokines were known as important factors that promoted the uncontrolled proliferation and migration in RPE cells [26]. The specific function of cytokines in the formation of PVR has become a research focus. Some studies showed that EGF contributed to the RPE cells’ proliferation and migration, and motivated the occurrence of PVR [8, 27, 28]. In this study, our results showed that the cells we isolated from eye tissues had the typical characteristics of epithelial cells. These cells shaped like an irregular hexagon and pebble, which consistent with the appearance of epithelial cells. At the same time, the isolated cells expressed the RPE characteristic proteins RPE65 and CRALBP. These results suggested that the isolated cells were human primary RPE cells. The fewer passages of the RPE cells, the better the specificity of the RPE cells are. However, the number of passages of the RPE cells is too small, the number of cells and the repeatability of the experiment are relatively poor. The RPE cells of passage 4 were relatively better in number, specificity, and biological authenticity. So, we detected expression of EGFR and survivin proteins in human RPE cells of passage 4 and ARPE-19 cell lines by immunoblotting and immunofluorescence. Our data indicated that EGFR and survivin proteins were exptessed in human RPE cells and ARPE-19 cell lines. These set the stage for further experiments. EGF was used to induce the EMT models in human primary RPE cells *in vitro*. Our results showed that EGF not only induced RPE cells to lose epithelial characteristics and transform to a spindle shape, but also significantly increased RPE cell vitality, proliferation, and migration. Additionally,EGF also activated phosphorylation of EGFR/MAPK proteins: EGFR, JNK, ERK, and P38, down regulated epithelial marker proteins: E-cadherin and ZO-1, and up regulated mesenchymal marker proteins: N-cadherin, a-SMA, and vimentin. These results indicated that EGF significantly resulted in EMT in RPE cells, which might be an effective model to simulate the pathological mechanism of PVR *in vitro*.

Survivin, a small protein composed of 42 amino acids also known as BIRC5, inhibits caspase activation and cell death and promotes cell division and growth [13]. Survivin expressed highly in tumor cells and expressed lower in normal cells, which makes it an important target for treating cancer [29]. Several studies have shown that survivin is involved in EMT mediated by multiple signaling pathways, including PI3K/AKT [30], Wnt/β-Catenin [31], and MAPK [32] signaling pathways. A previous study reported that EGF up-regulate the expression levels of survivin protein and activated the ERK signaling pathway [33]. Some researchers reported that survivin knockdown or inhibition affected TGFβ-induced EMT and significantly reduced cellular proliferation and migration in ARPE-19 cells [18]. Our previous research indicated that the survivin inhibitor YM155 inhibited cellular survival, proliferation and migration and resulted in cell death in ARPE-19 cells by EGFR/MAPK signaling [23]. In this study, our findings demonstrated that YM155 not only suppressed cellular viability, proliferation, and migration, but also mitigated the EGF-induced enhancement of cellular viability, proliferation, and migration in RPE cells. Survivin knockdown in RPE cells, achieved by transfection of survivin siRNA, inhibited cellular proliferation and migration and attenuated the EGF-induced increase of cellular proliferation and migration. Taken together, these results suggested survivin closely related to EGF-induced EMT of RPE cells and survivin inhibition might be a potential way to deal with RPE cells EMT in the process of PVR.

EGFR is an important membrane-bound receptor that is extensively expressed in many cells and has become a prominent target for therapeutic applications in some diseases and cancer therapy [34–36]. The interaction between EGFR and its ligand EGF played some pivotal roles in cell proliferation, differentiation, and migration [37, 38]. Some researcher reported that EGF could activate human RPE cells’ proliferation and migration by inducing the EGFR/MAPK signaling pathway in a concentration-dependent manner *in vitro* [39]. In this study, we obtained some similar results that EGF induced the phosphorylation of EGFR, JNK, ERK, and P38MAPK in a time-dependent manner, and caused an increase of RPE cells’ viability, proliferation, and migration. These results manifested that the EGF/EGFR/MAPK signaling pathway might promote the process of viability, proliferation, and migration of RPE cell. Some studies indicated that EGFR was closely related to survivin protein expression and EGF/EGFR signaling promoted survivin expression [40, 41]. It has been confirmed that the survivin inhibitor YM155 acted through the modulation of EGFR and survivin expression to reduce cancer cell survival [42]. Our previous study also showed that YM155 inhibited RPE cells’ survival through affecting the EGFR/MAPK pathway [23]. In this study, we found EGF activated EGFR/MAPK signaling pathways and simultaneously up-regulated the expression of survivin protein. The survivin inhibitor YM155 can suppress expression of the survivin protein, reduce the EGFR and phosphorylated ERK proteins, and increase pression levels of phosphorylated EGFR, JNK, and P38MAPK proteins. In addition, it is worth noting that survivin inhibition by using YM155 and survivin siRNA significantly attenuated EGF-induced phosphorylation of EGFR-MAPK. Taken together, our data showed targeting survivin protein and EGFR-MAPK signaling pathways could be an important way to inhibit abnormal RPE cell viability, proliferation, and migration. However, the exact molecular mechanism between the surviving and EGFR-MAPK signaling pathways remains to be confirmed by further experiments *in vivo*.

EMT is known as epithelial cells lose epithelial characteristics and translate into motile mesenchymal cells, which is a crucial mechanism in embryogenesis and tissue repair and contributes to the occurrence of such diseases as tissues fibrosis and cancer [43, 44]. Characteristic changes of the EMT include the following: a decrease of intercellular adhesion and the epithelial markers: E-cadherin and ZO-1; an increase of the mesenchymal markers: N-cadherin, α-SMA, and vimentin; and the acceleration of cell migration and invasion capacity [45]. It has been established that the EMT of REP cells is a key mechanism of PVR, although the pathological mechanisms of PVR remain unclear [46]. Some growth factors and cytokines, such as PDGF, TGF-β, EGF, TNF-α, have been used to induce EMT and investigate EMT markers in RPE cells [47]. Our results indicated that EGF induced RPE cells to lose their epithelial characteristics and to translate into mesenchymal-like cells; it also reduced the expression of E-cadherin and ZO-1 proteins, and increased the expression of N-cadherin, vimentin, and a-SMA proteins. As mentioned above, our results suggested the EMT in RPE cells was successfully induced by EGF in vitro. Some researchers found that YM155 reduced the expression levels of N-cadherin and vimentin proteins in ARPE-19 cells [18].

In this study, we not only found YM155 and survivin knockdown enhanced the expression levels of E-cadherin and ZO-1 proteins and reduced the expression of N-cadherin, vimentin, and a-SMA proteins, but also demonstrated that YM155 and survivin knockdown significantly attenuated EGF-induced a reduction of E-cadherin and ZO-1 proteins and an increase of N-cadherin, vimentin, and a-SMA in RPE cells. Although our results indicated that EGF resulted in EMT of RPE cells, and also revealed that survivin inhibition significantly affected EGF-induced changes of EMT-related proteins and abnormal proliferation and migration in RPE cells; it is not total certainty whether this function is valid *in vivo*, and it is essential to detect the specific mechanism and function of survivin inhibition through building some animal models of PVR.

**Fig 9:**
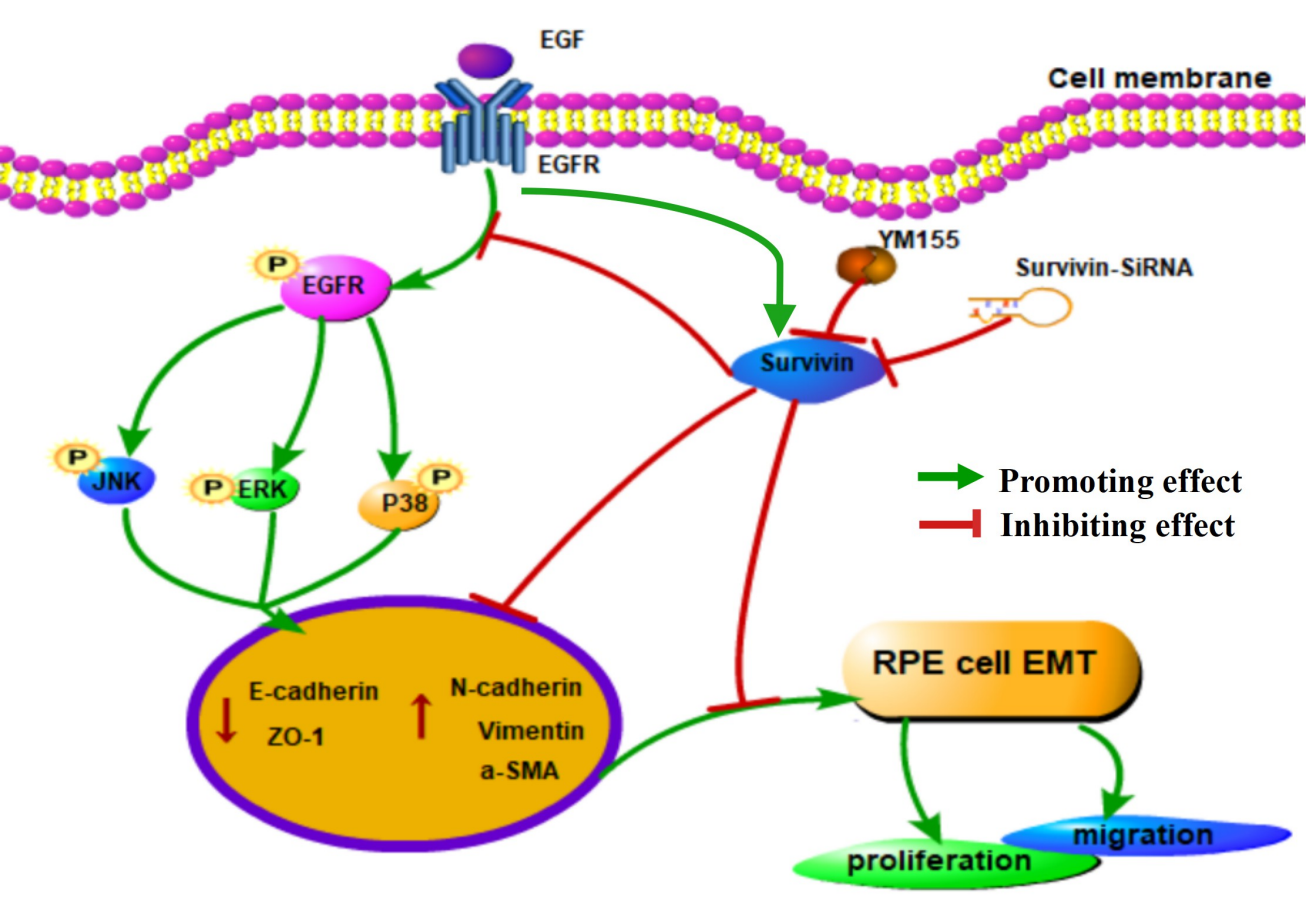
Schematic diagram of survivin inhibition affecting EGF-induced EMT in RPE cells via the EGFR/MAPK signaling pathway.

In conclusion, the present study takes advantage of EGF-induced the EMT model of RPE cells to explore the effects of survivin inhibition on the EGFR/MAPK proteins and some epithelial and mesenchymal marker proteins in the EMT process. It is clear that treatment with EGF resulted in obvious EMT in RPE cells by activating EGFR/MAPK signaling and survivin inhibition by using YM155 and survivin-siRNA suppressed EGF-induced EMT in RPE cells through the EGFR/MAPK pathway *in vitro* (Fig 9). Because EMT of RPE cells is a vital pathogenesis of PVR, our study might provide a useful model of EMT in RPE cells *in vitro* and supply a promising strategy for preventing and treating of PVR. Certainly, further basic animal experiments and clinical trials are essential.

## Supporting information

S1 File Raw images. (PDF)

S2 File Supporting information files. (DOCX)

S3 File Minimal data set. (PDF)
