# Supplementary figures and images for: Survivin inhibition attenuates EGF-induced epithelial mesenchymal transformation of human RPE cells via the EGFR/MAPK pathway
Source: PLoS One. 2024 Aug 30;19(8):e0309539. doi: 10.1371/journal.pone.0309539 (PMC11364297; doi:10.1371/journal.pone.0309539)

FIG.1

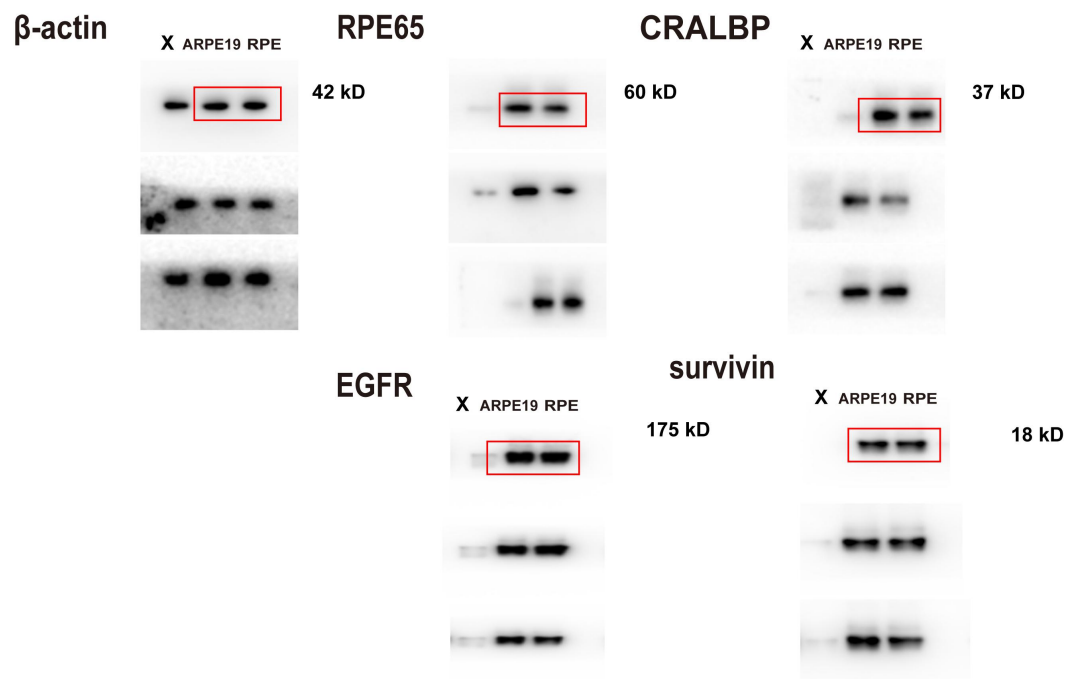

FIG.2-1

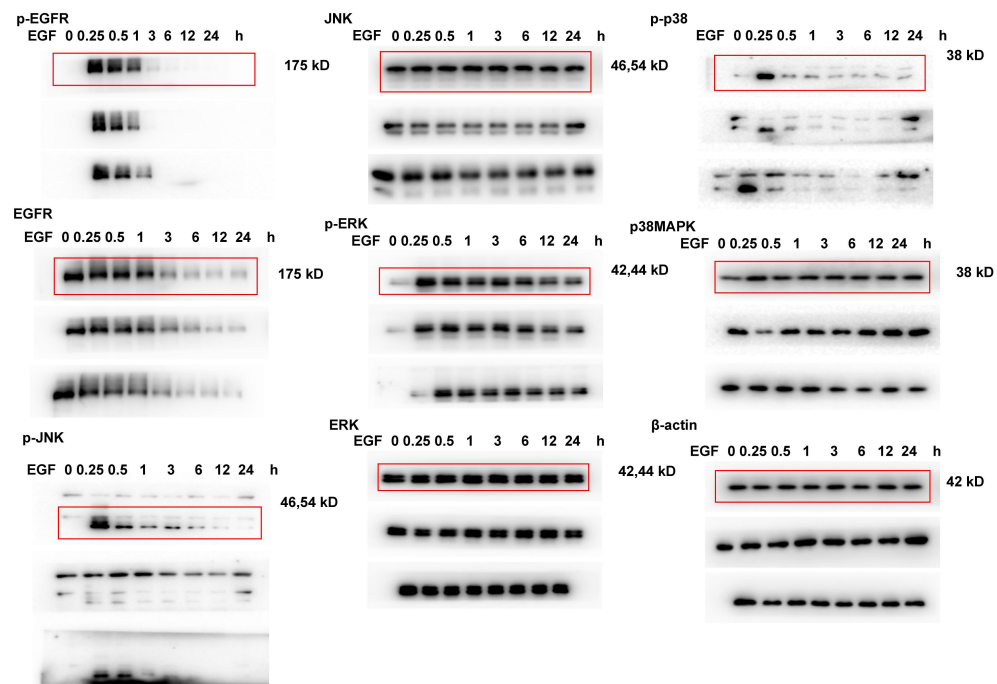

FIG.2-2

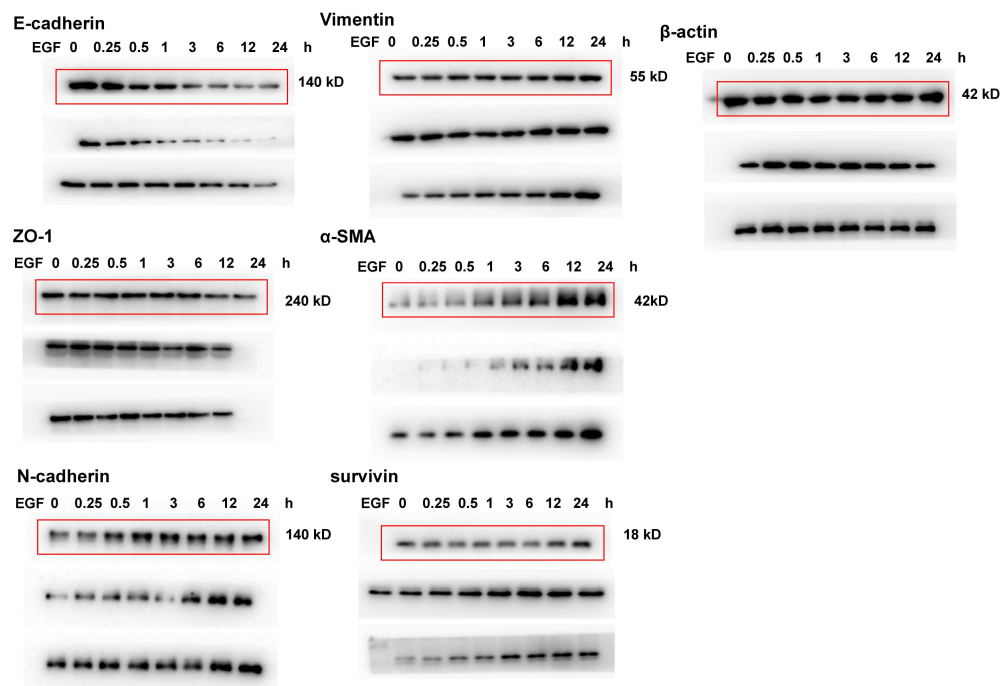

FIG.4-1

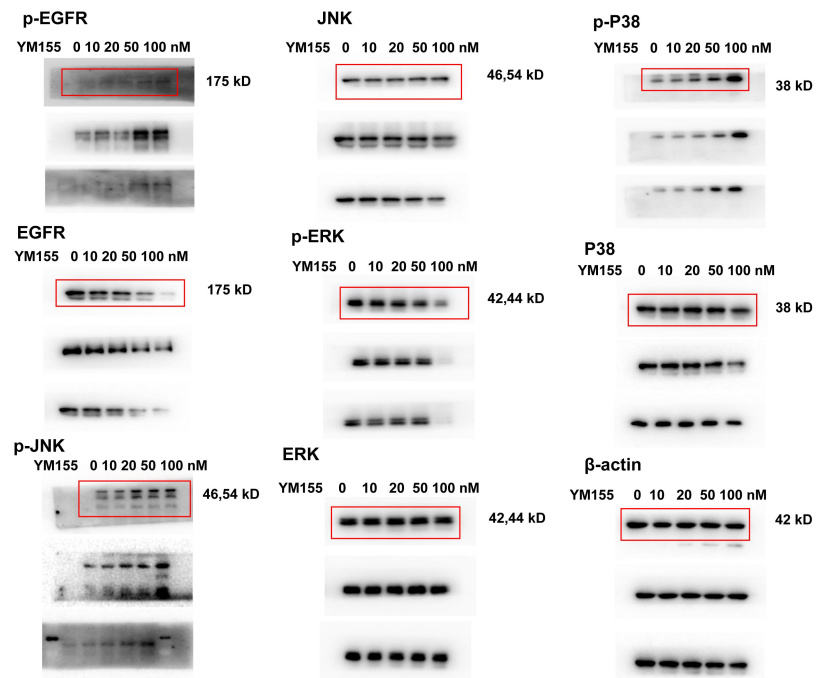

FIG.4-2

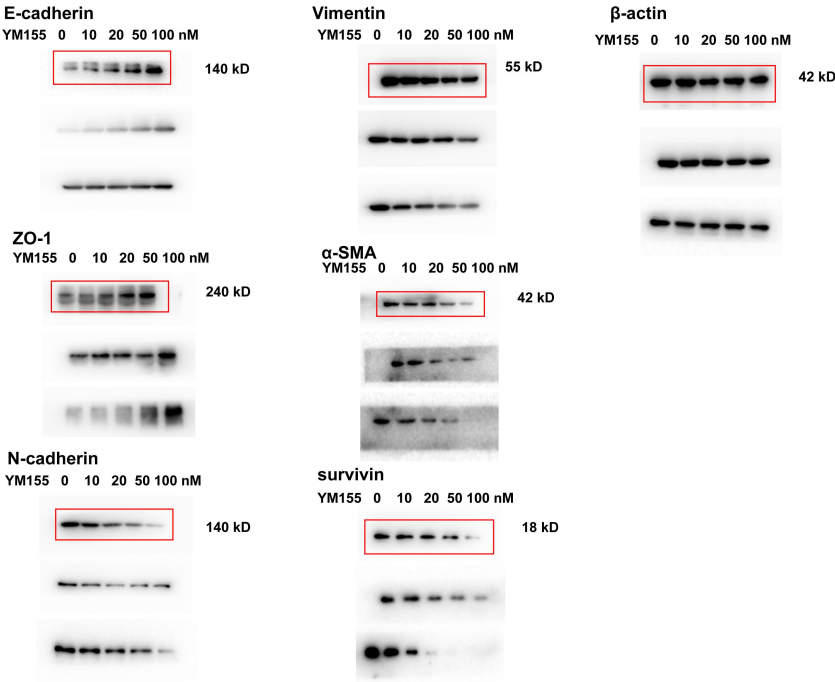

FIG.6-1

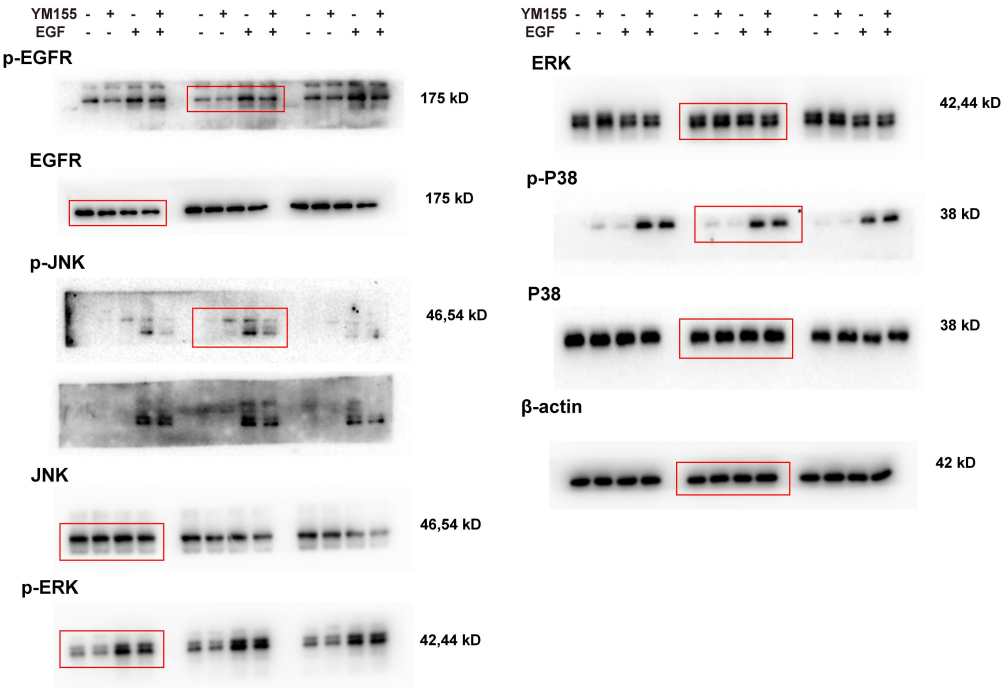

FIG.6-2

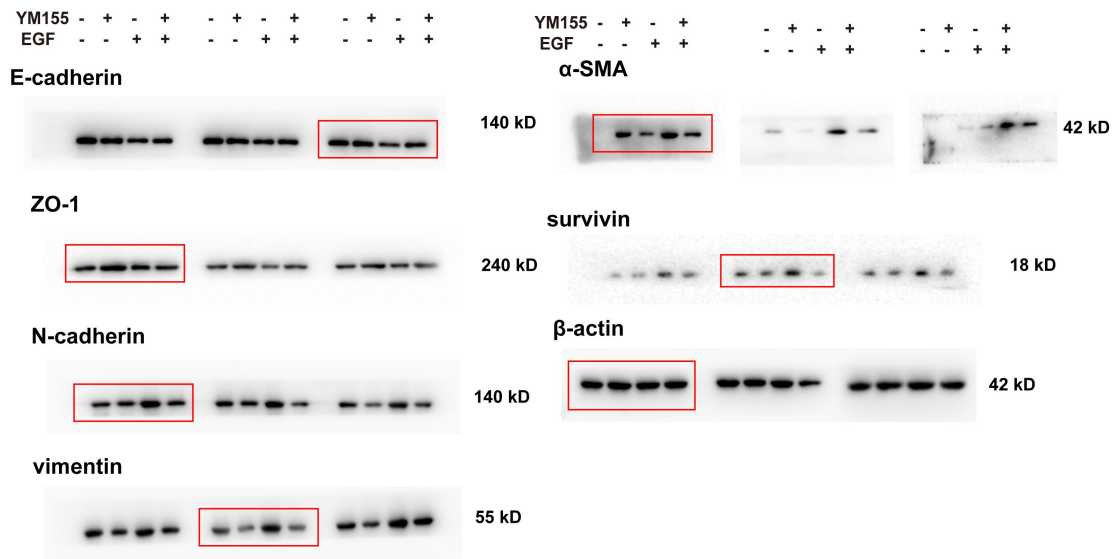

FIG.7-1

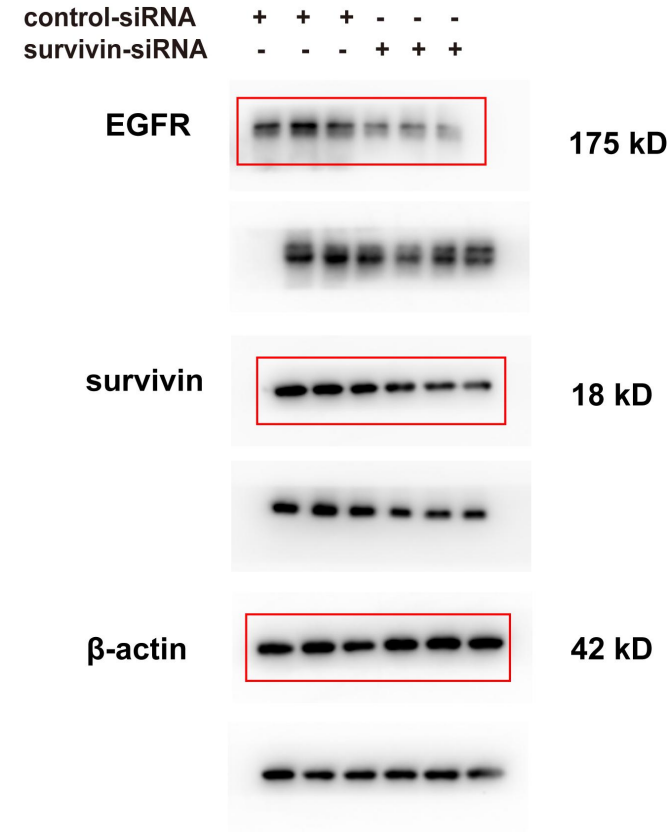

FIG.7-2

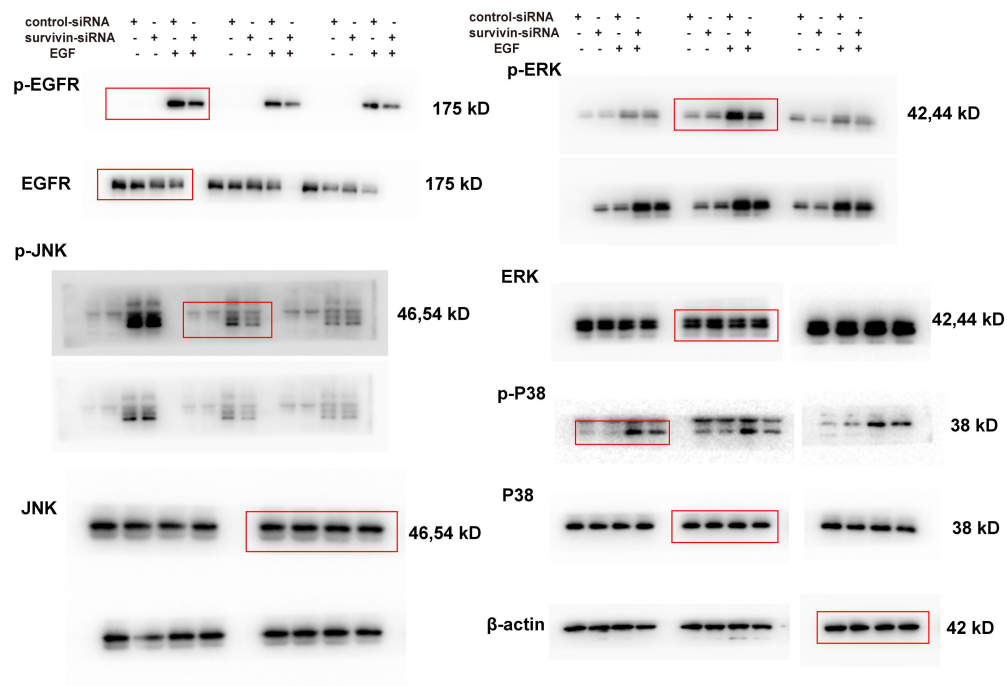

FIG.8-1

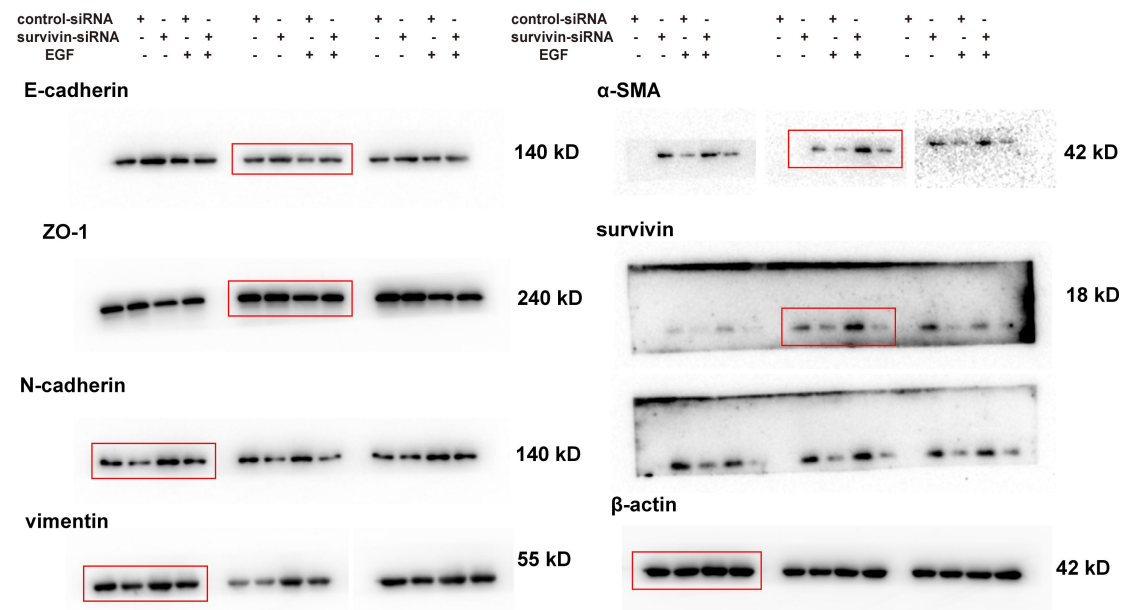

Supplement: S1 File — (PDF) [file pone.0309539.s001.pdf]
