# Supplementary material for: Survivin inhibition attenuates EGF-induced epithelial mesenchymal transformation of human RPE cells via the EGFR/MAPK pathway
Source: PLoS One. 2024 Aug 30;19(8):e0309539. doi: 10.1371/journal.pone.0309539 (PMC11364297; doi:10.1371/journal.pone.0309539)
Supplement: S2 File — (DOCX) [file pone.0309539.s002.docx]

FIG.1

A：


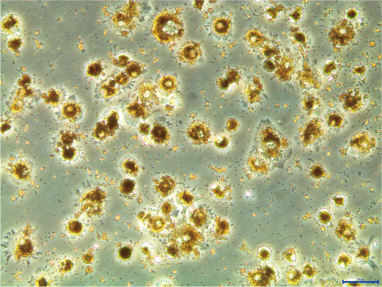

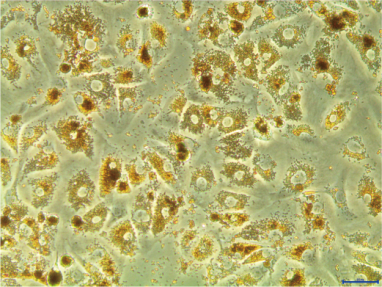


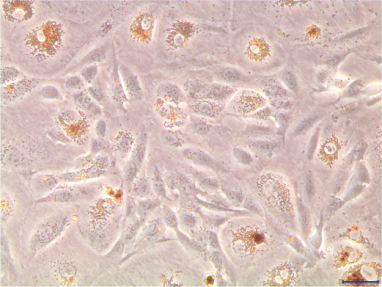

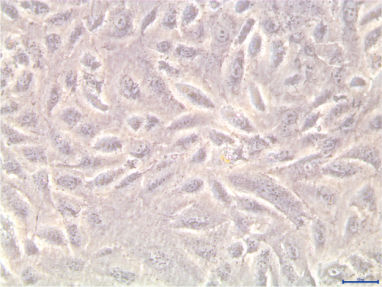


B:
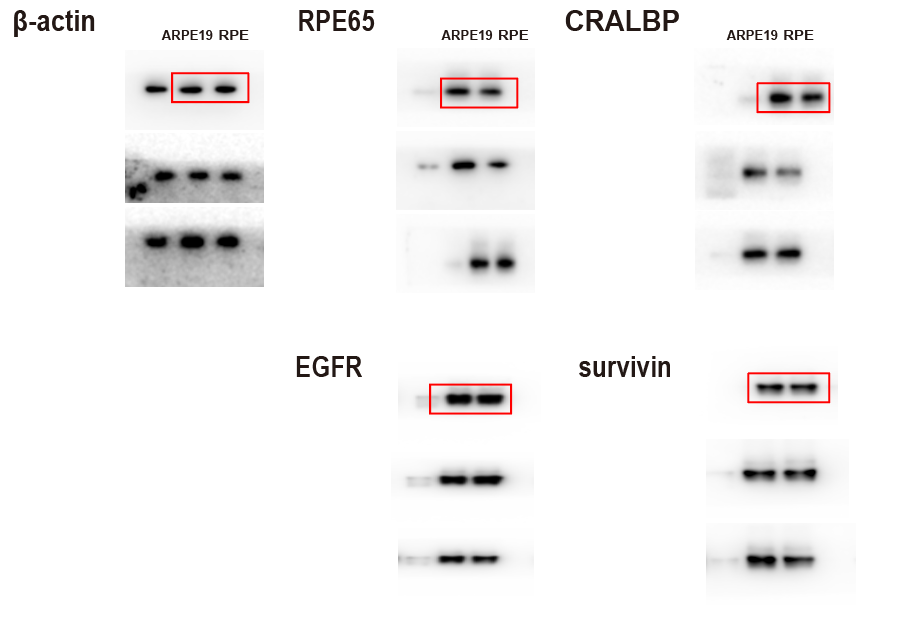


C:


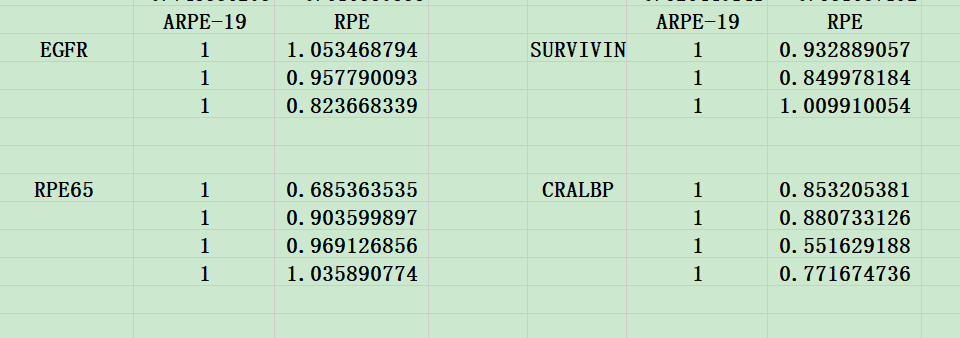


D:

RPE65


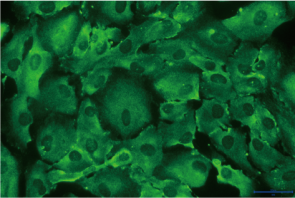

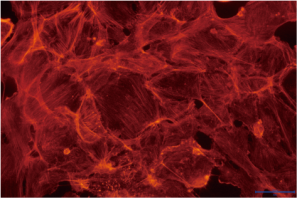

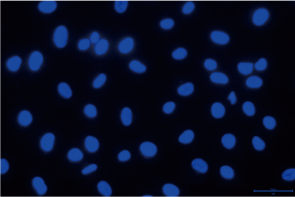

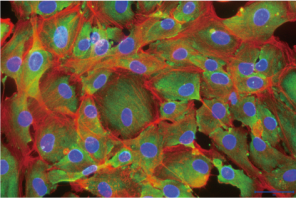


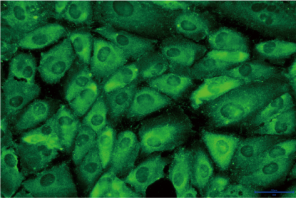

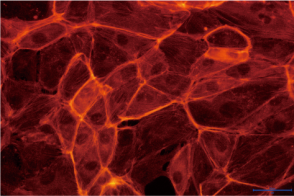

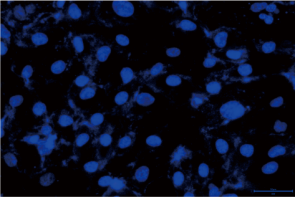

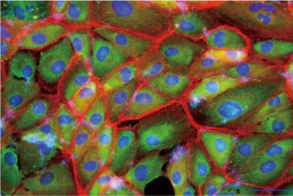


CRALBP


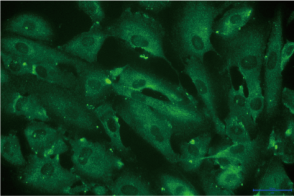

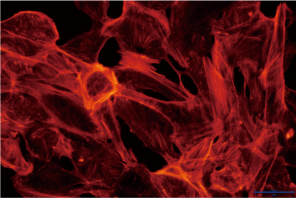

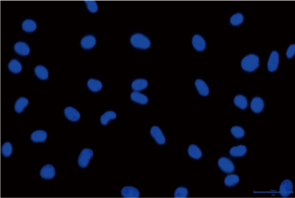

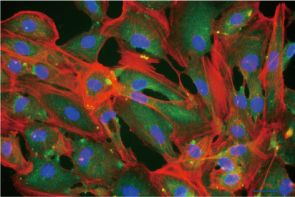


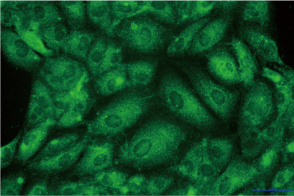

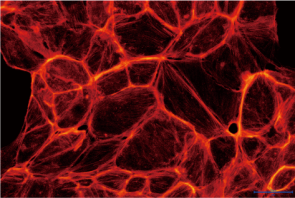

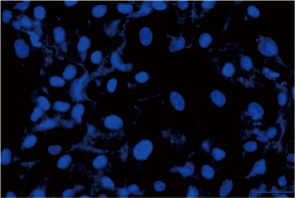

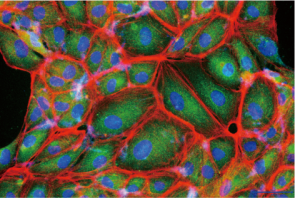


E

EGFR:


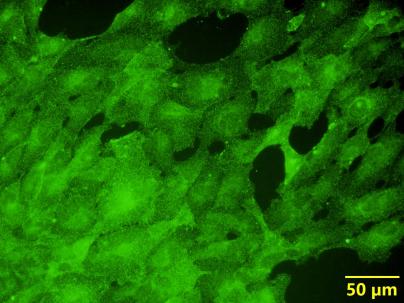

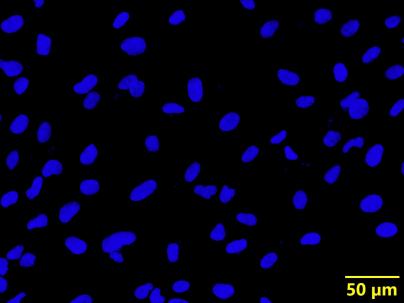

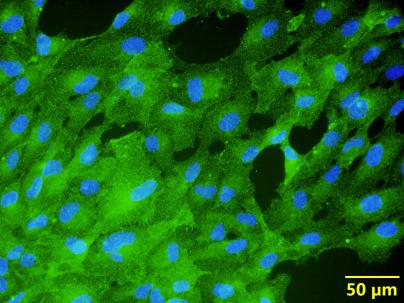


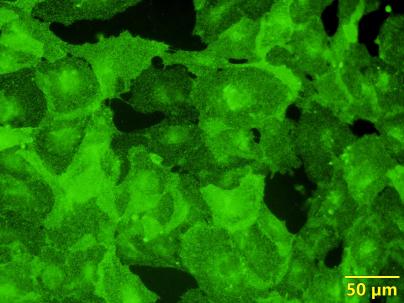

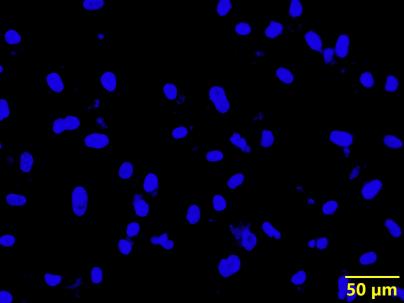

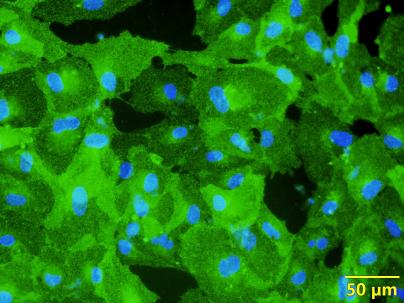


Survivin:


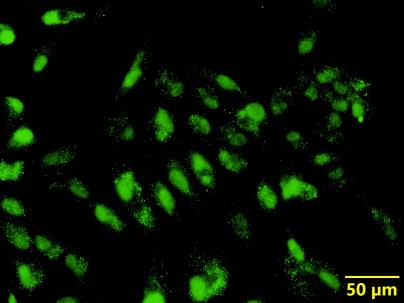

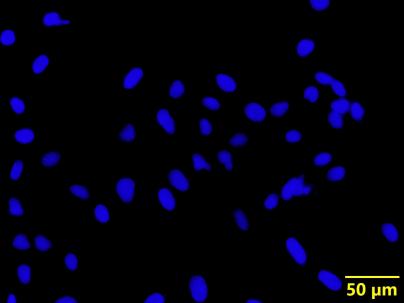

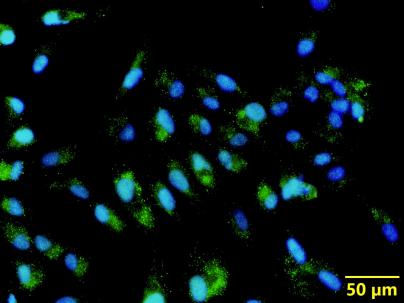


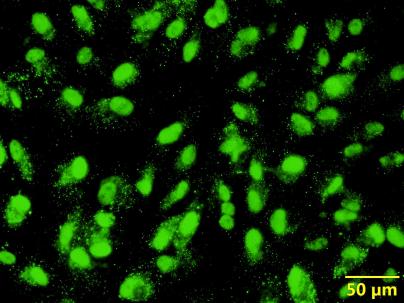

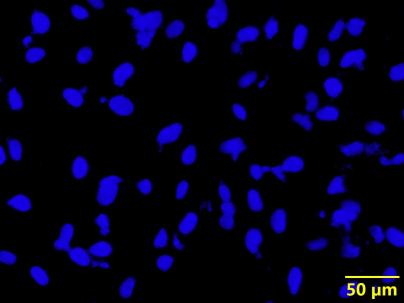

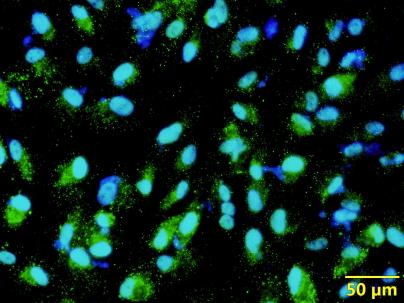


FIG.2

A: Control group和EGF group


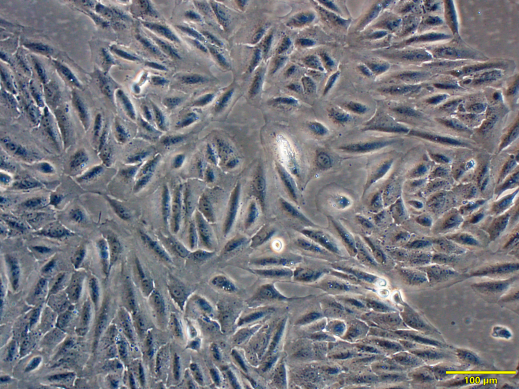

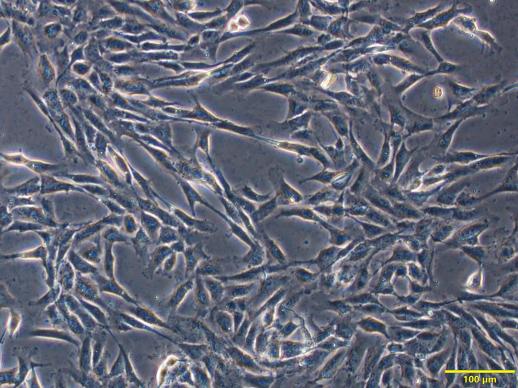


B:


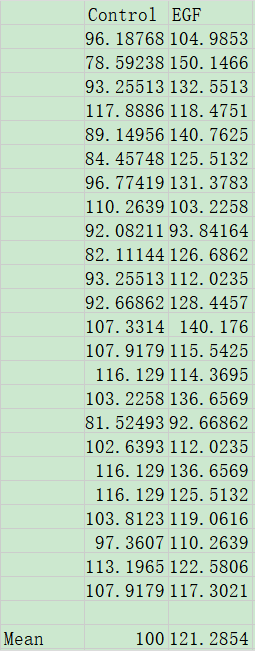


C:

Control group


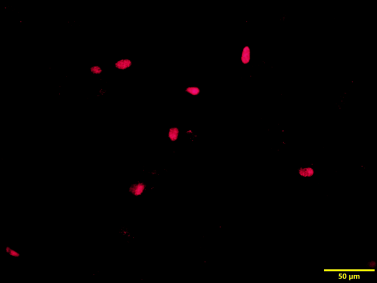

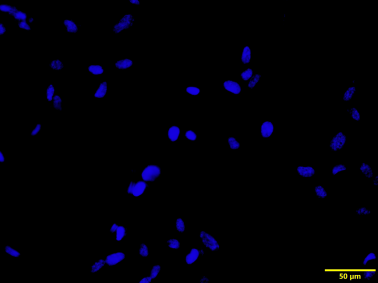

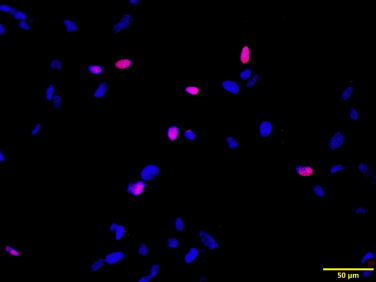


EGF group


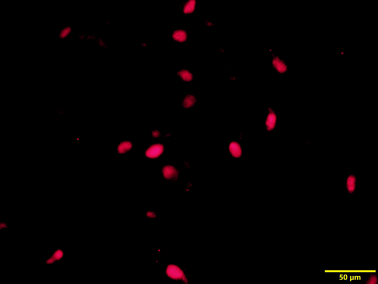

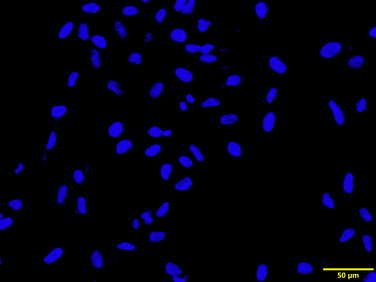

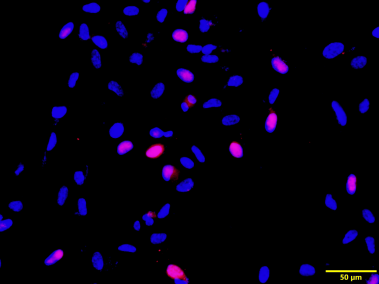


D：


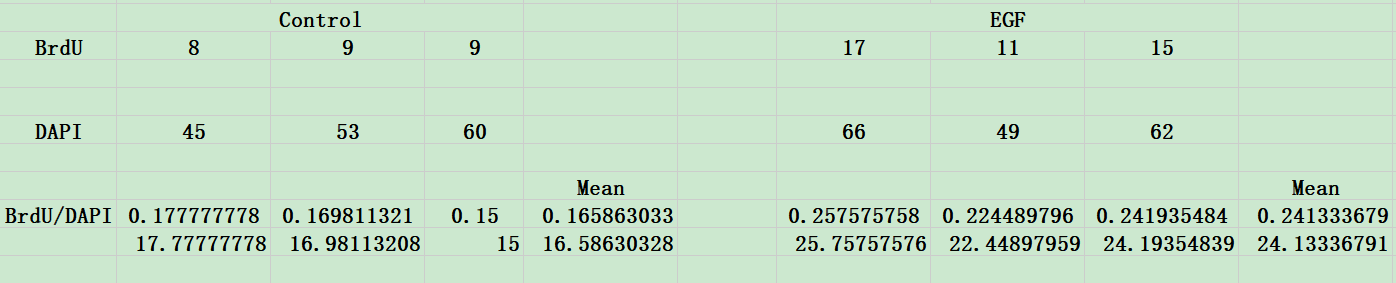


E:

Control group


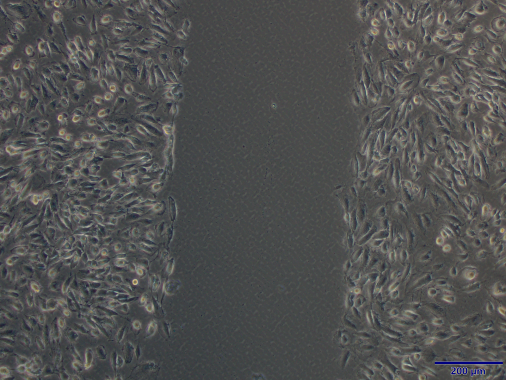

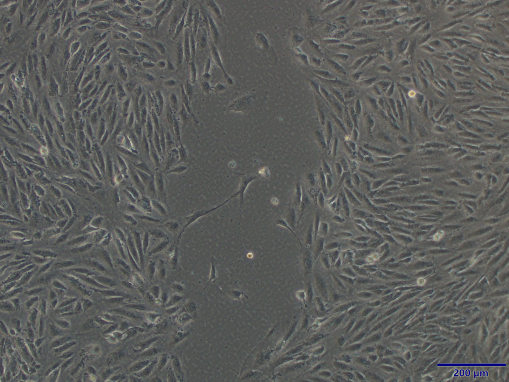


EGF group


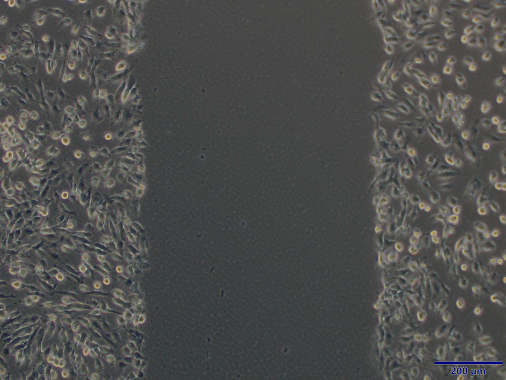

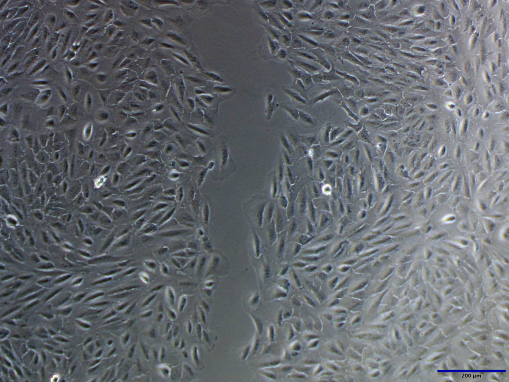


F：


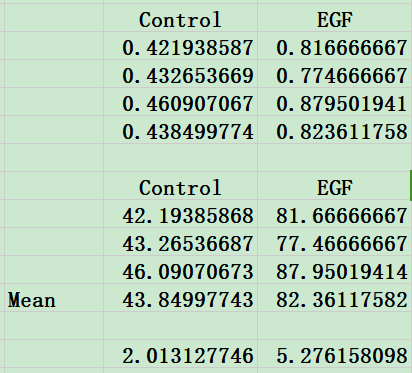


G:


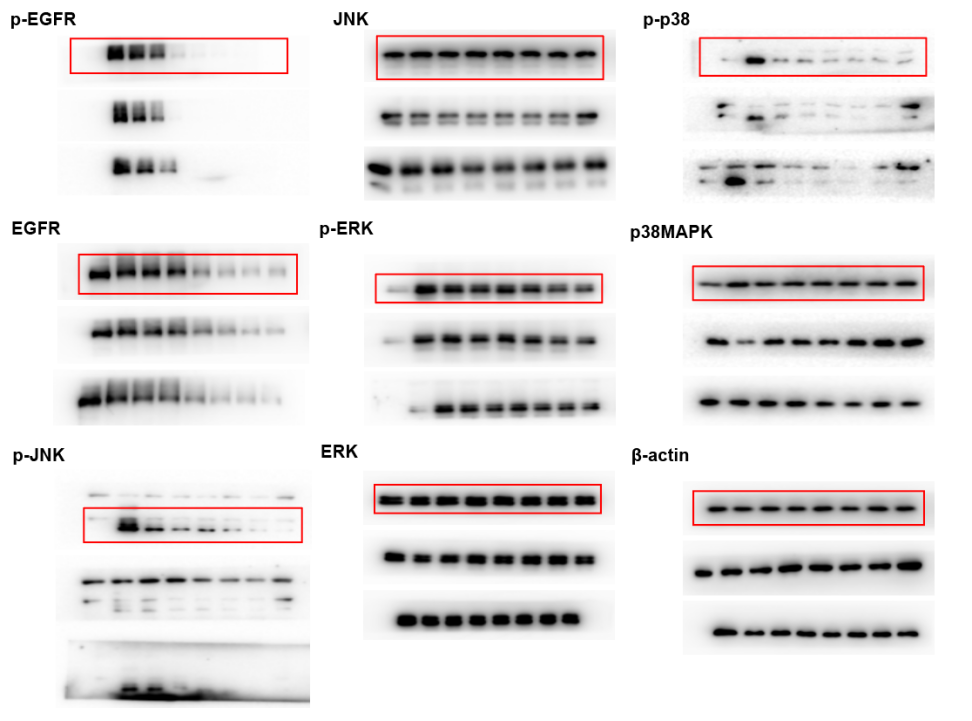


H:


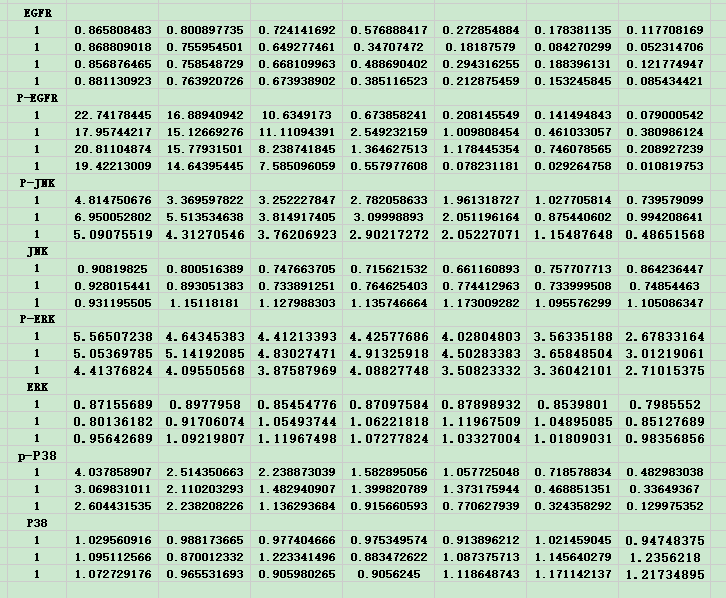


I:


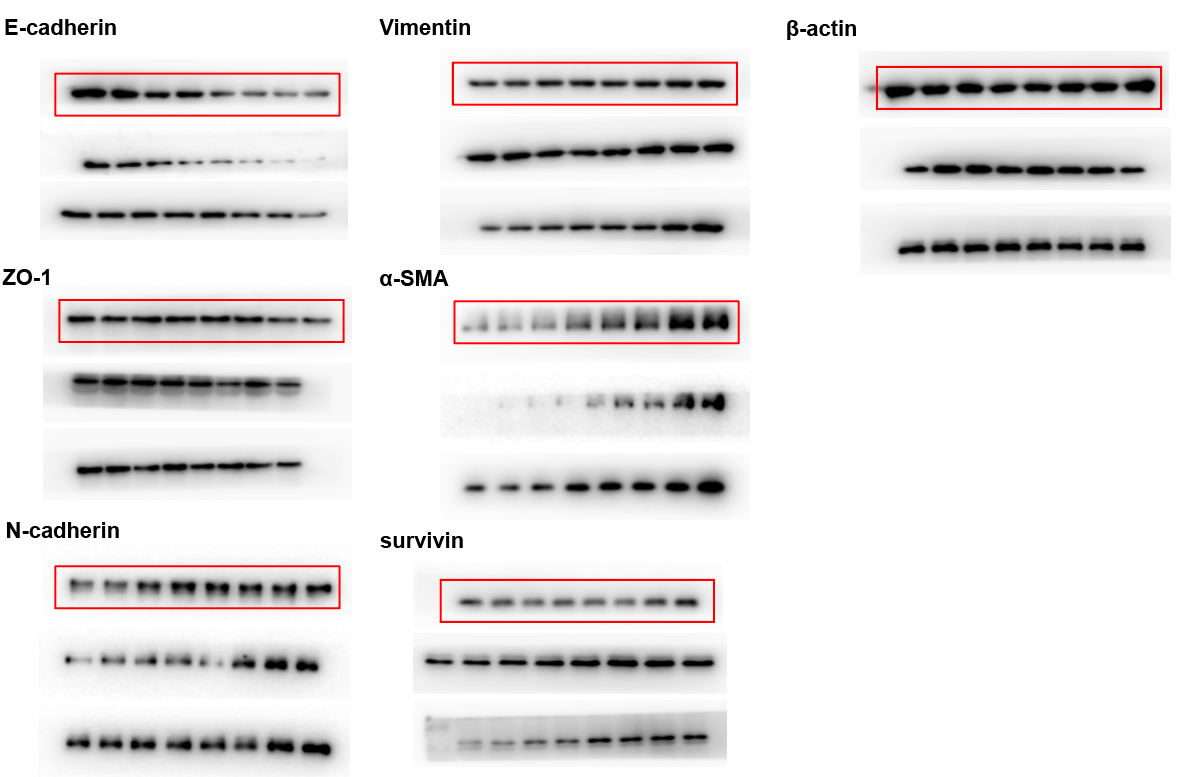


J:
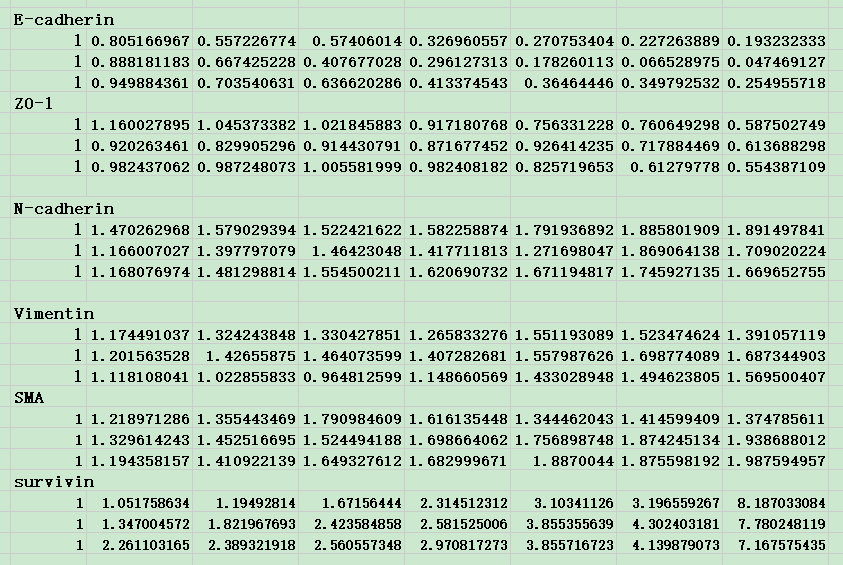


FIG.3

A：

Control group 6h，12h，24h


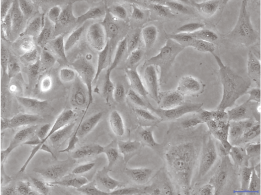

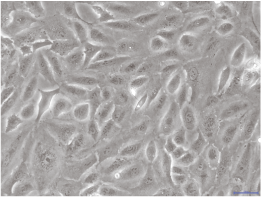

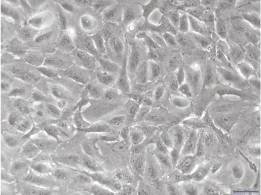


YM155 10nM group 6h，12h，24h


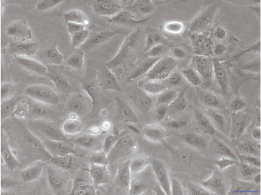

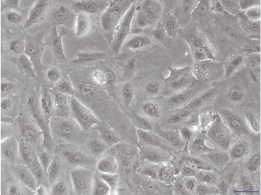

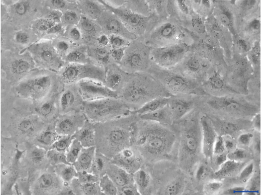


YM155 20nM group 6h，12h，24h


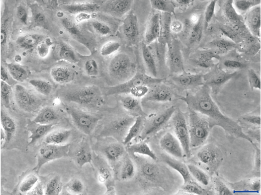

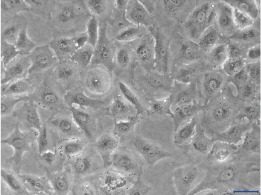

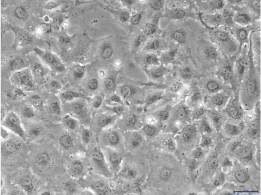


YM155 50nM group 6h，12h，24h


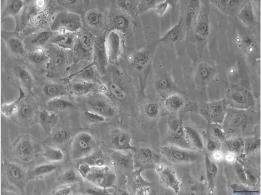

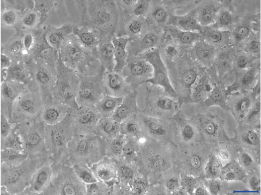

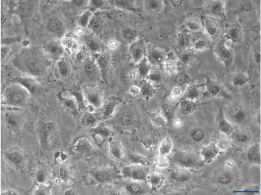


YM155 100nM group 6h，12h，24h


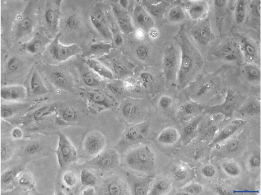

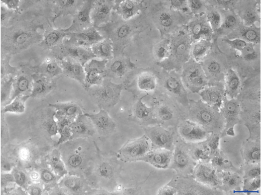

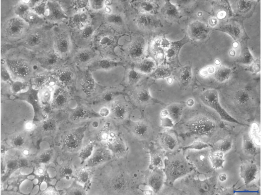


B:


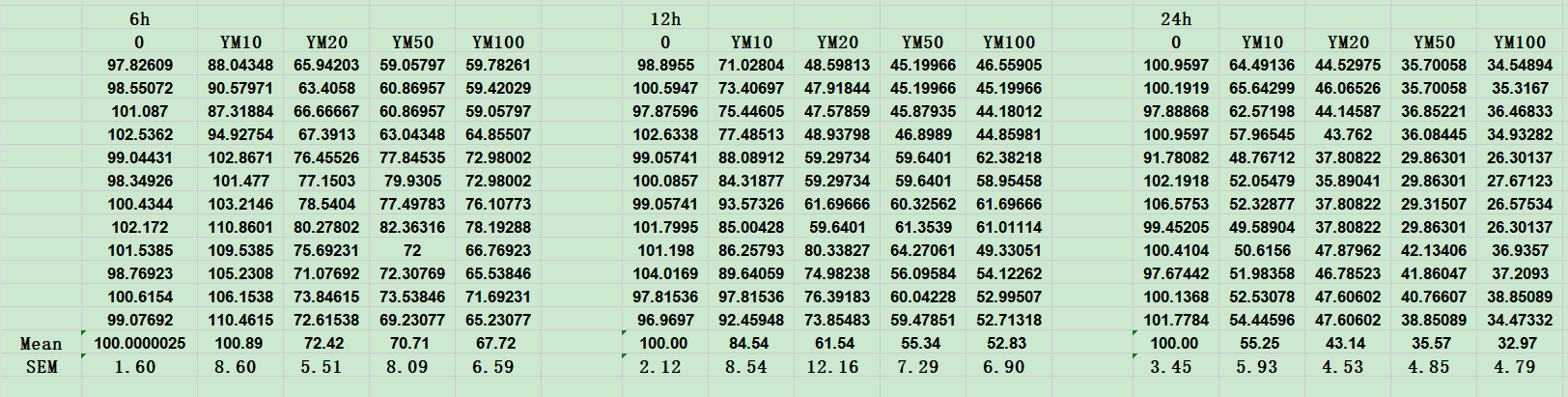


C:

Control group BrdU，DAPI，Merge


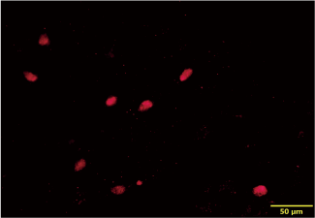

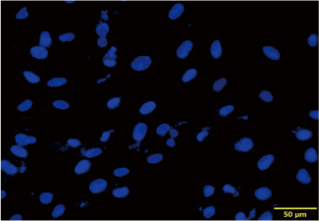

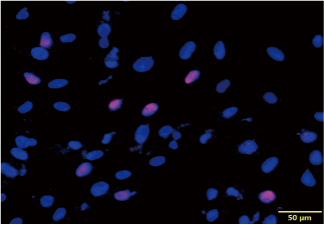


YM155 10nM group BrdU，DAPI，Merge


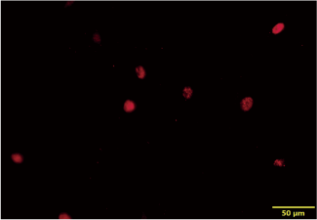

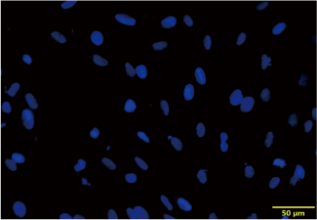

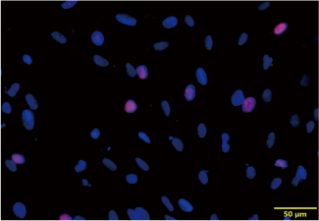


YM155 20nM group BrdU，DAPI，Merge


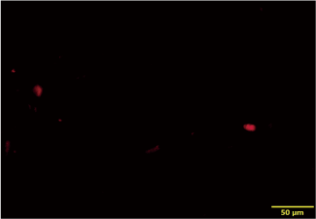

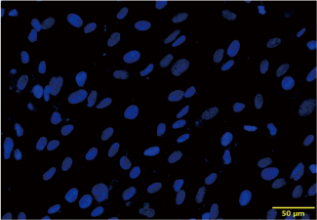

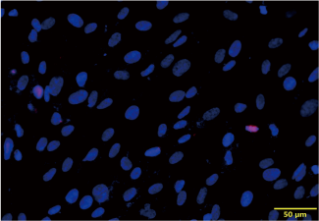


YM155 50nM group BrdU，DAPI，Merge


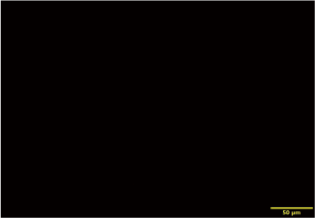

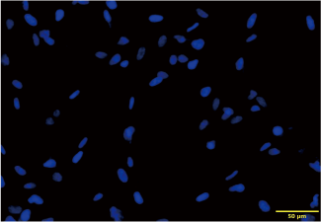

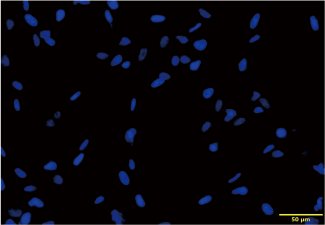


D:


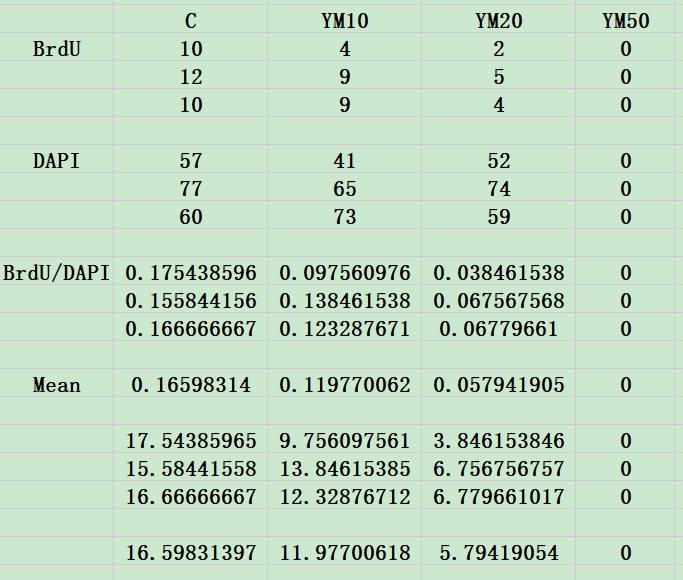


E:

Control group 0h,24h


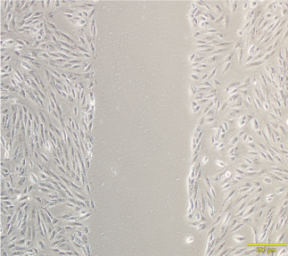

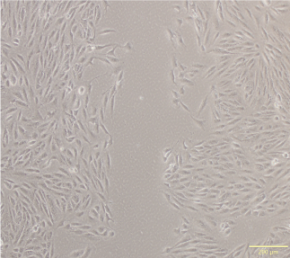


YM155 10nM group 0h,24h


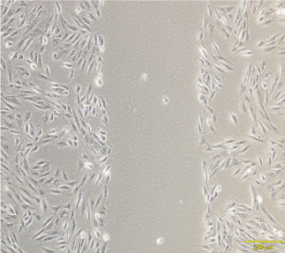

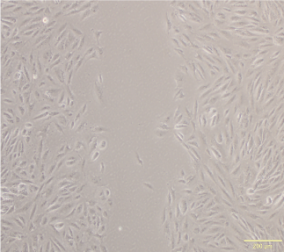


YM155 20nM group 0h,24h


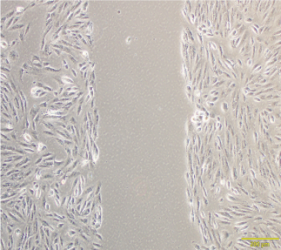

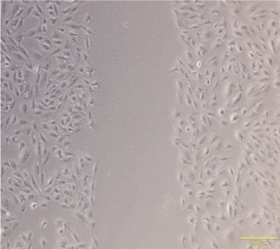


YM155 50nM group 0h,24h


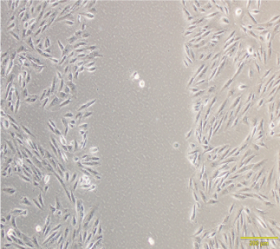

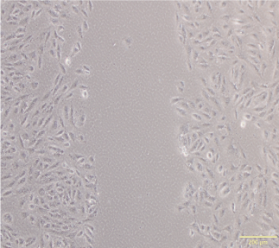


F:


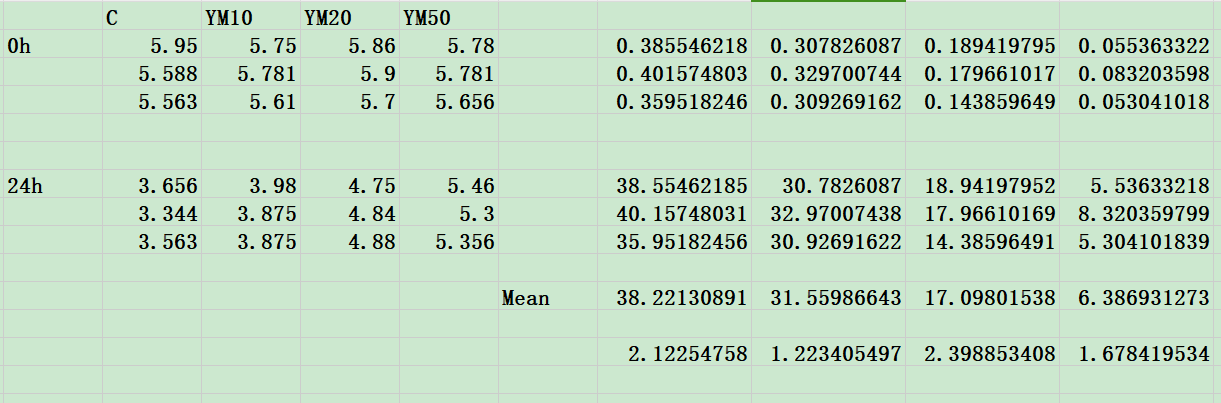


FIG.4

A:


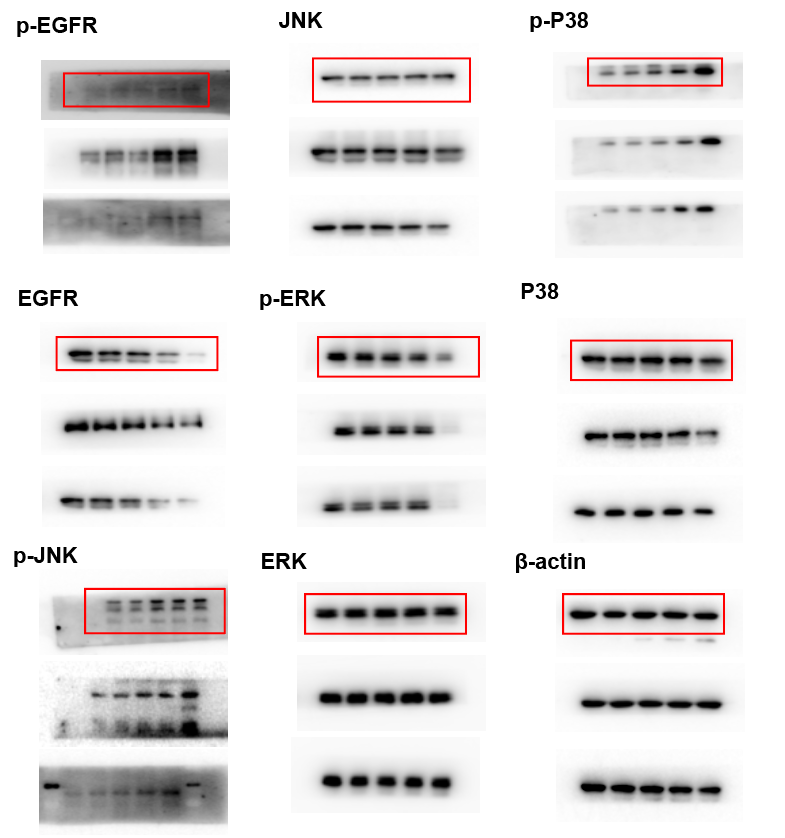


B:


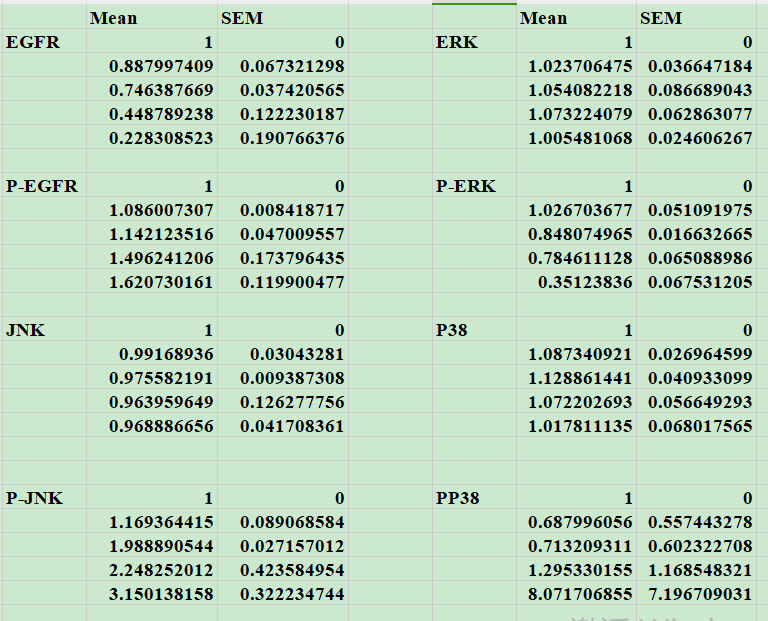


C:

Control group p-EGFR


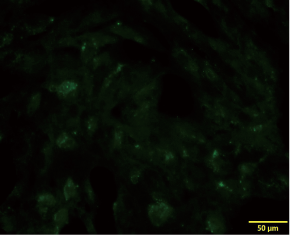

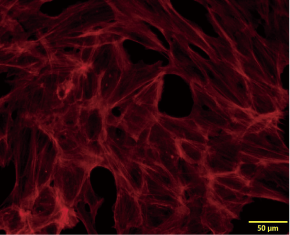

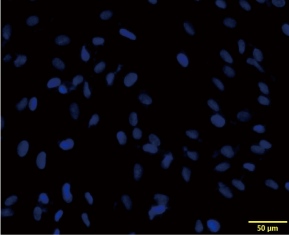

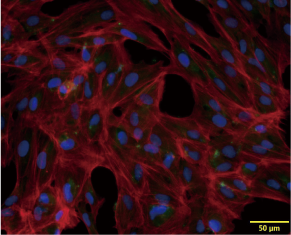


100nM YM155 group p-EGFR


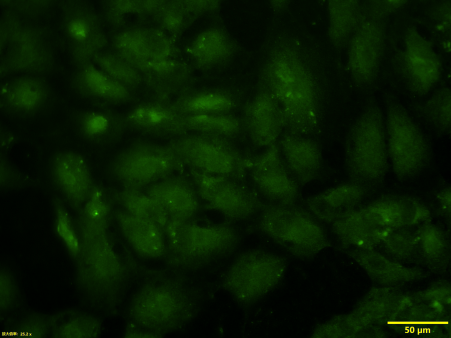

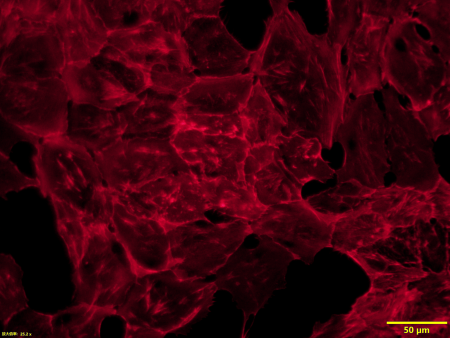


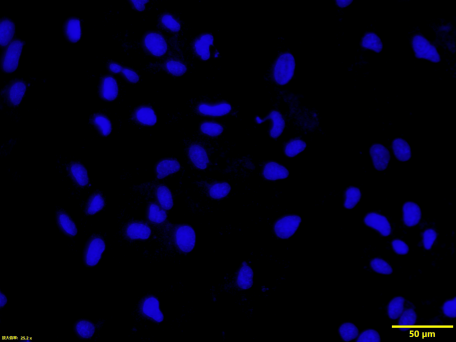


Control group EGFR

100nM YM155 group EGFR

D：

E：

FIG.5

A:

Controlgroup：

20nM YM155group：

100ng/mL EGFgroup：

YM155+EGFgroup：

B：

C:

0h Control group、20nM YM155 group、EGF group、YM155+EGF group：

24h Control group、20nM YM155group、EGF group、YM155+EGF group：

D:

FIG.6

A:

B:

C:

p-EGFR, Control group

p-EGFR, 20nM YM155 group

p-EGFR, EGF group

p-EGFR, YM155+EGF group

EGFR, Control group

EGFR, 20 nM YM155 group

EGFR, EGF group

EGFR, YM155+EGF group

D:

E:

FIG.7

A:

B:

C:

si-control group：

si-survivin group：

si-control+EGF group：

si-survivin+EGF group

D:

E：

0h si-control, si-survivin, si-control+EGF, si-survivin+EGF:

24h si-control, si-survivin, si-control+EGF, si-survivin+EGF:

F:

G:

H:

FIG.8

A:

B:

C:

E-cadherin:

ZO-1:

1. cadherin:

α-SMA:
