# Supplementary material for: Survivin inhibition attenuates EGF-induced epithelial mesenchymal transformation of human RPE cells via the EGFR/MAPK pathway
Source: PLoS One. 2024 Aug 30;19(8):e0309539. doi: 10.1371/journal.pone.0309539 (PMC11364297; doi:10.1371/journal.pone.0309539)
Supplement: S3 File — (PDF) [file pone.0309539.s003.pdf]

FIG.1C

	[illegible]	[illegible]		[illegible]	[illegible]
	ARPE-19	RPE		ARPE-19	RPE
EGFR	1	1.053468794	SURVIVIN	1	0.932889057
	1	0.957790093		1	0.849978184
	1	0.823668339		1	1.009910054
RPE65	1	0.685363535	CRALBP	1	0.853205381
	1	0.903599897		1	0.880733126
	1	0.969126856		1	0.551629188
	1	1.035890774		1	0.771674736

FIG.2B

	Control	EGF
	96.18768	104.9853
	78.59238	150.1466
	93.25513	132.5513
	117.8886	118.4751
	89.14956	140.7625
	84.45748	125.5132
	96.77419	131.3783
	110.2639	103.2258
	92.08211	93.84164
	82.11144	126.6862
	93.25513	112.0235
	92.66862	128.4457
	107.3314	140.176
	107.9179	115.5425
	116.129	114.3695
	103.2258	136.6569
	81.52493	92.66862
	102.6393	112.0235
	116.129	136.6569
	116.129	125.5132
	103.8123	119.0616
	97.3607	110.2639
	113.1965	122.5806
	107.9179	117.3021
Mean	100	121.2854

FIG.2D

		Control					EGF		
BrdU	8	9	9			17	11	15	
DAPI	45	53	60			66	49	62	
				Mean					Mean
BrdU/DAPI	0.177777778	0.169811321	0.15	0.165863033		0.257575758	0.224489796	0.241935484	0.241333679
	17.77777778	16.98113208	15	16.58630328		25.75757576	22.44897959	24.19354839	24.13336791

FIG.2F

	Control	EGF
	0.421938587	0.816666667
	0.432653669	0.774666667
	0.460907067	0.879501941
	0.438499774	0.823611758
	Control	EGF
	42.19385868	81.66666667
	43.26536687	77.46666667
	46.09070673	87.95019414
Mean	43.84997743	82.36117582
	2.013127746	5.276158098

FIG.2H

EGFR							
1	0.865808483	0.800897735	0.724141692	0.576888417	0.272854884	0.178381135	0.117708169
1	0.868809018	0.755954501	0.649277461	0.34707472	0.18187579	0.084270299	0.052314706
1	0.856876465	0.758548729	0.668109963	0.488690402	0.294316255	0.188396131	0.121774947
1	0.881130923	0.763920726	0.673938902	0.385116523	0.212875459	0.153245845	0.085434421
P-EGFR							
1	22.74178445	16.88940942	10.6349173	0.673858241	0.208145549	0.141494843	0.079000542
1	17.95744217	15.12669276	11.11094391	2.549232159	1.009808454	0.461033057	0.380986124
1	20.81104874	15.77931501	8.238741845	1.364627513	1.178445354	0.746078565	0.208927239
1	19.42213009	14.64395445	7.585096059	0.557977608	0.078231181	0.029264758	0.010819753
P-JNK							
1	4.814750676	3.369597822	3.252227847	2.782058633	1.961318727	1.027705814	0.739579099
1	6.950052802	5.513534638	3.814917405	3.09998893	2.051196164	0.875440602	0.994208641
1	5.09075519	4.31270546	3.76206923	2.90217272	2.05227071	1.15487648	0.48651568
JNK							
1	0.90819825	0.800516389	0.747663705	0.715621532	0.661160893	0.757707713	0.864236447
1	0.928015441	0.893051383	0.733891251	0.764625403	0.774412963	0.733999508	0.74854463
1	0.931195505	1.15118181	1.127988303	1.135746664	1.173009282	1.095576299	1.105086347
P-ERK							
1	5.56507238	4.64345383	4.41213393	4.42577686	4.02804803	3.56335188	2.67833164
1	5.05369785	5.14192085	4.83027471	4.91325918	4.50283383	3.65848504	3.01219061
1	4.41376824	4.09550568	3.87587969	4.08827748	3.50823332	3.36042101	2.71015375
ERK							
1	0.87155689	0.8977958	0.85454776	0.87097584	0.87898932	0.8539801	0.7985552
1	0.80136182	0.91706074	1.05493744	1.06221818	1.11967509	1.04895085	0.85127689
1	0.95642689	1.09219807	1.11967498	1.07277824	1.03327004	1.01809031	0.98356856
p-P38							
1	4.037858907	2.514350663	2.238873039	1.582895056	1.057725048	0.718578834	0.482983038
1	3.069831011	2.110203293	1.482940907	1.399820789	1.373175944	0.468851351	0.33649367
1	2.604431535	2.238208226	1.136293684	0.915660593	0.770627939	0.324358292	0.129975352
P38							
1	1.029560916	0.988173665	0.977404666	0.975349574	0.913896212	1.021459045	0.94748375
1	1.095112566	0.870012332	1.223341496	0.883472622	1.087375713	1.145640279	1.2356218
1	1.072729176	0.965531693	0.905980265	0.9056245	1.118648743	1.171142137	1.21734895

FIG.2J

E-cadherin							
1	0.805166967	0.557226774	0.57406014	0.326960557	0.270753404	0.227263889	0.193232333
1	0.888181183	0.667425228	0.407677028	0.296127313	0.178260113	0.066528975	0.047469127
1	0.949884361	0.703540631	0.636620286	0.413374543	0.36464446	0.349792532	0.254955718
ZO-1							
1	1.160027895	1.045373382	1.021845883	0.917180768	0.756331228	0.760649298	0.587502749
1	0.920263461	0.829905296	0.914430791	0.871677452	0.926414235	0.717884469	0.613688298
1	0.982437062	0.987248073	1.005581999	0.982408182	0.825719653	0.61279778	0.554387109
N-cadherin							
1	1.470262968	1.579029394	1.522421622	1.582258874	1.791936892	1.885801909	1.891497841
1	1.166007027	1.397797079	1.46423048	1.417711813	1.271698047	1.869064138	1.709020224
1	1.168076974	1.481298814	1.554500211	1.620690732	1.671194817	1.745927135	1.669652755
Vimentin							
1	1.174491037	1.324243848	1.330427851	1.265833276	1.551193089	1.523474624	1.391057119
1	1.201563528	1.42655875	1.464073599	1.407282681	1.557987626	1.698774089	1.687344903
1	1.118108041	1.022855833	0.964812599	1.148660569	1.433028948	1.494623805	1.569500407
SMA							
1	1.218971286	1.355443469	1.790984609	1.616135448	1.344462043	1.414599409	1.374785611
1	1.329614243	1.452516695	1.524494188	1.698664062	1.756898748	1.874245134	1.938688012
1	1.194358157	1.410922139	1.649327612	1.682999671	1.8870044	1.875598192	1.987594957
survivin							
1	1.051758634	1.19492814	1.67156444	2.314512312	3.10341126	3.196559267	8.187033084
1	1.347004572	1.821967693	2.423584858	2.581525006	3.855355639	4.302403181	7.780248119
1	2.261103165	2.389321918	2.560557348	2.970817273	3.855716723	4.139879073	7.167575435

FIG.3B

	6h				
	0	YM10	YM20	YM50	YM100
	97.82609	88.04348	65.94203	59.05797	59.78261
	98.55072	90.57971	63.4058	60.86957	59.42029
	101.087	87.31884	66.66667	60.86957	59.05797
	102.5362	94.92754	67.3913	63.04348	64.85507
	99.04431	102.8671	76.45526	77.84535	72.98002
	98.34926	101.477	77.1503	79.9305	72.98002
	100.4344	103.2146	78.5404	77.49783	76.10773
	102.172	110.8601	80.27802	82.36316	78.19288
	101.5385	109.5385	75.69231	72	66.76923
	98.76923	105.2308	71.07692	72.30769	65.53846
	100.6154	106.1538	73.84615	73.53846	71.69231
	99.07692	110.4615	72.61538	69.23077	65.23077
Mean	100.0000025	100.89	72.42	70.71	67.72
SEM	1.60	8.60	5.51	8.09	6.59

	12h				
	0	YM10	YM20	YM50	YM100
	98.8955	71.02804	48.59813	45.19966	46.55905
	100.5947	73.40697	47.91844	45.19966	45.19966
	97.87596	75.44605	47.57859	45.87935	44.18012
	102.6338	77.48513	48.93798	46.8989	44.85981
	99.05741	88.08912	59.29734	59.6401	62.38218
	100.0857	84.31877	59.29734	59.6401	58.95458
	99.05741	93.57326	61.69666	60.32562	61.69666
	101.7995	85.00428	59.6401	61.3539	61.01114
	101.198	86.25793	80.33827	64.27061	49.33051
	104.0169	89.64059	74.98238	56.09584	54.12262
	97.81536	97.81536	76.39183	60.04228	52.99507
	96.9697	92.45948	73.85483	59.47851	52.71318
Mean	100.00	84.54	61.54	55.34	52.83
SEM	2.12	8.54	12.16	7.29	6.90

	24h				
	0	YM10	YM20	YM50	YM100
	100.9597	64.49136	44.52975	35.70058	34.54894
	100.1919	65.64299	46.06526	35.70058	35.3167
	97.88868	62.57198	44.14587	36.85221	36.46833
	100.9597	57.96545	43.762	36.08445	34.93282
	91.78082	48.76712	37.80822	29.86301	26.30137
	102.1918	52.05479	35.89041	29.86301	27.67123
	106.5753	52.32877	37.80822	29.31507	26.57534
	99.45205	49.58904	37.80822	29.86301	26.30137
	100.4104	50.6156	47.87962	42.13406	36.9357
	97.67442	51.98358	46.78523	41.86047	37.2093
	100.1368	52.53078	47.60602	40.76607	38.85089
	101.7784	54.44596	47.60602	38.85089	34.47332
Mean	100.00	55.25	43.14	35.57	32.97
SEM	3.45	5.93	4.53	4.85	4.79

FIG.3D

	C	YM10	YM20	YM50
BrdU	10	4	2	0
	12	9	5	0
	10	9	4	0
DAPI	57	41	52	0
	77	65	74	0
	60	73	59	0
BrdU/DAPI	0.175438596	0.097560976	0.038461538	0
	0.155844156	0.138461538	0.067567568	0
	0.166666667	0.123287671	0.06779661	0
Mean	0.16598314	0.119770062	0.057941905	0
	17.54385965	9.756097561	3.846153846	0
	15.58441558	13.84615385	6.756756757	0
	16.66666667	12.32876712	6.779661017	0
	16.59831397	11.97700618	5.79419054	0

FIG.3F

	C	YM10	YM20	YM50					
0h	5.95	5.75	5.86	5.78		0.385546218	0.307826087	0.189419795	0.055363322
	5.588	5.781	5.9	5.781		0.401574803	0.329700744	0.179661017	0.083203598
	5.563	5.61	5.7	5.656		0.359518246	0.309269162	0.143859649	0.053041018
24h	3.656	3.98	4.75	5.46		38.55462185	30.7826087	18.94197952	5.53633218
	3.344	3.875	4.84	5.3		40.15748031	32.97007438	17.96610169	8.320359799
	3.563	3.875	4.88	5.356		35.95182456	30.92691622	14.38596491	5.304101839
					Mean	38.22130891	31.55986643	17.09801538	6.386931273
						2.12254758	1.223405497	2.398853408	1.678419534

FIG.4B

	Mean	SEM			Mean	SEM
EGFR	1	0		ERK	1	0
	0.887997409	0.067321298			1.023706475	0.036647184
	0.746387669	0.037420565			1.054082218	0.086689043
	0.448789238	0.122230187			1.073224079	0.062863077
	0.228308523	0.190766376			1.005481068	0.024606267
P-EGFR	1	0		P-ERK	1	0
	1.086007307	0.008418717			1.026703677	0.051091975
	1.142123516	0.047009557			0.848074965	0.016632665
	1.496241206	0.173796435			0.784611128	0.065088986
	1.620730161	0.119900477			0.35123836	0.067531205
JNK	1	0		P38	1	0
	0.99168936	0.03043281			1.087340921	0.026964599
	0.975582191	0.009387308			1.128861441	0.040933099
	0.963959649	0.126277756			1.072202693	0.056649293
	0.968886656	0.041708361			1.017811135	0.068017565
P-JNK	1	0		PP38	1	0
	1.169364415	0.089068584			0.687996056	0.557443278
	1.988890544	0.027157012			0.713209311	0.602322708
	2.248252012	0.423584954			1.295330155	1.168548321
	3.150138158	0.322234744			8.071706855	7.196709031

FIG.4E

0	10	20	50	100		0	10	20	50	100
E-cadherin						Vimentin				
1	1.795709159	2.381736552	3.340832852	3.860599971		1	0.946105411	0.815633535	0.751424307	0.519397186
1	1.850193031	3.001918436	4.510139533	6.175915262		1	0.931887153	0.721888641	0.7624396	0.697366239
1	1.224923053	1.201253007	1.334748747	1.513785069		1	1.062511642	0.805607829	0.746358171	0.777757876
ZO-1						SMA				
1	1.211545721	1.45671564	1.87887059	2.089016091		1	0.946105411	0.815633535	0.751424307	0.519397186
1	1.187395553	1.273132872	1.595954667	1.951122891		1	0.931887153	0.721888641	0.7624396	0.490614291
1	1.15543082	1.394551409	1.654367403	1.791426249		1	1.062511642	0.805607829	0.746358171	0.750591409
N-cadherin						survivin				
1	0.70186953	0.367994661	0.464336691	0.302347679		1	0.941368826	0.779244097	0.597488952	0.150664258
1	0.808987861	0.61510446	0.392146688	0.135785539		1	1.007570298	0.597067296	0.52531691	0.351129883
1	0.905143814	0.709503646	0.630883258	0.214663027		1	0.912167597	0.789150992	0.58870946	0.470694005

FIG.5B

	Control	YM155	EGF	YM155+EGF
BrdU/DAPI	0.170731707	0.096774194	0.263157895	0.122807018
	0.183098592	0.116666667	0.235294118	0.137931034
	0.151515152	0.112676056	0.296875	0.122807018
	17.07317073	9.677419355	26.31578947	12.28070175
	18.30985915	11.66666667	23.52941176	13.79310345
	15.15151515	11.26760563	29.6875	12.28070175
Mean	16.84484835	10.87056389	26.51090041	12.78483565
		5.974284461	9.666052067	13.72606476
SEM	1.591503234	1.052381979	3.083677008	0.873185525

FIG.5D

	C	YM155	EGF	Y+E
0H	0.454126679	0.164682927	0.653205742	0.408238969
	0.439176426	0.172571429	0.727962963	0.337372207
	0.437383178	0.112092658	0.682855471	0.358454838
24H	45.41266795	16.46829268	65.32057416	40.82389692
	43.91764261	17.25714286	72.7962963	33.73722075
	43.73831776	11.2092658	68.2855471	35.84548381
Mean	44.35620944	14.97823378	68.80080585	36.80220049
		29.37797566	24.44459641	31.99860536
SEM	0.919302879	3.287766851	3.764402215	3.638918091

FIG.6B

	Control	YM155	EGF	Y+E			Control	YM155	EGF	Y+E
p-EGFR	1	0.924114393	19.55332525	1.361507505		p-ERK	1	0.79110049	1.860700174	1.261422573
	1	1.17999614	24.9794002	2.371847026			1	0.783177696	1.965013036	1.022444246
	1	0.966913708	17.17610686	1.163413812			1	0.750593564	1.893383394	0.99686202
	1	1.042419113	15.63532362	1.260581492						
	1	0.785669627	20.38843591	0.715196793		ERK	1	0.947853567	0.914530248	0.88227115
	1	1.309950514	19.17510775	1.279179265			1	0.989427396	1.06222823	1.066158979
							1	1.109423457	0.890586617	0.857227029
EGFR	1	0.891080913	0.751686753	0.53156014						
	1	0.889490122	0.748418027	0.63209256		p-P38	1	1.044050312	7.069931826	4.663276064
	1	0.88995599	0.773413969	0.623285977			1	0.929485326	5.447183914	4.594009982
							1	1.644584049	6.188762619	3.200096747
p-JNK	1	1.877305856	4.856389443	1.926874278						
	1	2.68207885	5.54939304	2.617318255		P38	1	1.045444102	0.972430298	0.906817475
	1	2.327963222	5.185666677	2.248964101			1	1.082036997	1.072861182	1.166523735
							1	1.089131508	0.889899415	0.986550094
JNK	1	0.934149102	0.82473908	0.881557688						
	1	0.938105196	1.06851151	0.821805931						
	1	0.775576242	0.814545105	0.73861718						

FIG.6E

	Control	YM155	EGF	Y+E			Control	YM155	EGF	Y+E
E-cad	1	1.383644253	0.60187035	0.854262079		Vim	1	0.729075317	1.663548911	0.768417507
	1	1.351197948	0.680874172	0.772872448			1	0.790193763	1.845328783	0.746430013
	1	1.328510513	0.675239367	0.891206259			1	0.704354375	1.743476814	0.753906527
ZO-1	1	1.38056546	0.83948058	0.972607404		SMA	1	0.259078364	3.293184572	1.700569788
	1	1.34023163	0.705043332	1.040829764			1	0.373349841	2.043242971	1.530180648
	1	1.354308807	0.887475844	1.051145969			1	0.254555644	3.18150426	1.856678048
							1	0.11423221	2.619712692	1.499984462
N-cad	1	0.754748174	1.62089298	1.202164297						
	1	0.68162698	1.554071729	0.710613392		survivin	1	0.583105667	2.086419881	0.666595184
	1	0.787553155	1.440636833	1.06261814			1	0.586074964	1.659356313	0.479566851
	1	0.657702392	1.447111883	0.895186413			1	0.545620299	1.636101468	0.691634854

FIG.7B

	con-siRNA	si-survivin		con-siRNA	si-survivin
EGFR	22165.853	12141.518		1	0.547757761
	26465.225	12982.004		1	0.490530649
	22652.539	10355.782		1	0.457157672
SURVIVIN	28330.974	19294.075		1	0.681024062
	26439.861	17256.296		1	0.65266213
	21933.276	13085.832		1	0.59662004

FIG.7D

	C	KD	C+EGF	KD+EGF
BrdU	8	5	16	7
	9	5	12	7
	9	5	16	6
DAPI	49	51	51	73
	55	73	52	54
	48	53	61	46
BrdU/DAPI	0.1632653	0.09803922	0.31372549	0.09589041
	0.1636364	0.06849315	0.23076923	0.12962963
	0.1875	0.09433962	0.26229508	0.13043478
	0.1714672	0.08695733	0.26892993	0.11865161
	16.326531	9.80392157	31.372549	9.5890411
	16.363636	6.84931507	23.0769231	12.962963
	18.75	9.43396226	26.2295082	13.0434783
Mean	17.146722	8.69573297	26.8929934	11.8651608
		8.45098936	9.74627111	15.0278327
SEM	1.3886031	1.6097086	4.18742307	1.97158851

FIG.7F

	C	KD	C+EGF	KD+EGF
	0.453074434	0.327118644	0.679153094	0.4464
	0.458143075	0.3171875	0.69183359	0.554012346
	0.418181818	0.279674797	0.746192893	0.525889968
	C	KD	C+EGF	KD+EGF
	45.30744337	32.71186441	67.91530945	44.64
	45.81430746	31.71875	69.18335901	55.40123457
	41.81818182	27.96747967	74.61928934	52.58899676
Mean	44.31331088	30.79936469	70.5726526	50.87674378
		13.51394619	26.25934172	19.69590882
SEM	2.175656127	2.502248714	3.561381629	5.581209553

FIG.7H

	Si-CON	Si-SURVIVIN	Si-CON+EGF	Si-SURVIVIN+EGF		Si-CON	Si-SURVIVIN	Si-CON+EGF	i-SURVIVIN+EGF
p-EGFR	1	1.029987224	23.14949844	7.214713258	P-ERK	1	1.550253194	4.15791347	2.471946672
	1	0.737651916	15.68996122	4.783094656		1	1.304988717	3.048200155	2.19148505
	1	1.365834518	17.55795428	7.163927387		1	0.98421626	2.893949374	2.043575735
EGFR	1	0.773708723	0.381313576	0.294994702	ERK	1	1.11492471	1.156635773	1.105473888
	1	0.723585412	0.712722699	0.494464664		1	1.057127107	0.841986614	0.907040654
	1	0.751756531	0.699800495	0.510727298		1	0.992781998	0.915690418	0.943092595
	1	0.760209684	0.45660347	0.078284573					
	1	0.785410195	0.670135719	0.369547354	P-P38	1	1.239538365	3.283894028	1.823564332
						1	0.898946697	1.631979391	1.16388132
P-JNK	1	1.422671681	3.463064712	2.409255182		1	1.528001864	3.681856834	1.757286123
	1	1.134659543	3.554955495	1.953119361		1	0.862810008	2.748724327	1.484320775
	1	1.654912704	3.69570814	1.999711186					
					P38	1	1.020985876	0.974954412	0.99369262
JNK	1	1.021251206	1.020470182	0.937835901		1	1.098560475	1.110464164	0.963057844
	1	0.93378919	0.839215946	0.95551262		1	1.015933047	1.137646232	0.931903984
	1	1.043944771	0.994884167	0.907609076					

FIG.8B

	Si-CON	Si-SURVIVIN	Si-CON+EGF	Si-SURVIVIN+EGF		Si-CON	Si-SURVIVIN	Si-CON+EGF	Si-SURVIVIN+EGF
E-cadherin	1	1.379229209	0.291567955	1.856309981	Vimentin	1	0.71619904	1.43633698	0.837680932
	1	1.483600263	0.308118155	1.651710665		1	0.716427765	1.543684977	0.856546676
	1	1.682074146	0.302048321	1.55107114		1	0.662425427	1.566488152	0.810768877
	1	1.595688813	0.262348343	1.4085575		1	0.743546547	1.679672818	0.845391944
ZO-1	1	1.111555544	0.841545412	0.913870792	SMA	1	0.591503047	2.031979214	0.6003285
	1	1.151296139	0.853993452	0.970285218		1	0.457048816	1.527894474	0.436523888
	1	1.065748124	0.802650207	0.922151381		1	0.424872919	1.447649273	0.362257914
N-cadherin	1	0.629173854	1.521603671	0.740223976	Survivin	1	0.571286087	1.307476137	0.319451375
	1	0.695858586	1.114824279	0.524584038		1	0.460571222	1.322928634	0.405555235
	1	0.664618443	1.602593013	0.658121011		1	0.585952205	1.439481986	0.454319821
	1	0.645342822	1.97668461	0.568637229					
	1	0.640128981	1.385525129	0.687573283					
